# Pathogen Induced Changes in the Protein Profile of Human Tears from *Fusarium* Keratitis Patients

**DOI:** 10.1371/journal.pone.0053018

**Published:** 2013-01-08

**Authors:** Sivagnanam Ananthi, Namperumalsamy Venkatesh Prajna, Prajna Lalitha, Murugesan Valarnila, Kuppamuthu Dharmalingam

**Affiliations:** 1 Dr. G. Venkataswamy Eye Research Institute, Aravind Medical Research Foundation, Aravind Eye Care System, Madurai, India; 2 Cornea Clinic, Aravind Eye Hospital, Aravind Eye Care System, Madurai, India; 3 Department of Microbiology, Aravind Eye Hospital, Aravind Eye Care System, Madurai, India; 4 School of Biotechnology, Madurai Kamaraj University, Madurai, India; New York State Health Department and University at Albany, United States of America

## Abstract

*Fusarium* is the major causative agent of fungal infections leading to corneal ulcer (keratitis) in Southern India and other tropical countries. Keratitis caused by *Fusarium* is a difficult disease to treat unless antifungal therapy is initiated during the early stages of infection. In this study tear proteins were prepared from keratitis patients classified based on the duration of infection. Among the patients recruited, early infection (n = 35), intermediate (n = 20), late (n = 11), samples from five patients in each group were pooled for analysis. Control samples were a pool of samples from 20 patients. Proteins were separated on difference gel electrophoresis (DIGE) and the differentially expressed proteins were quantified using DeCyder software analysis. The following differentially expressed proteins namely alpha-1-antitrypsin, haptoglobin α2 chain, zinc-alpha-2-glycoprotein, apolipoprotein, albumin, haptoglobin precursor - β chain, lactoferrin, lacrimal lipocalin precursor, cystatin SA III precursor, lacritin precursor were identified using mass spectrometry. Variation in the expression level of some of the proteins was confirmed using western blot analysis. This is the first report to show stage specific tear protein profile in fungal keratitis patients. Validation of this data using a much larger sample set could lead to clinical application of these findings.

## Introduction

Corneal ulceration is the most common cause of corneal blindness in developing countries [1]. A retrospective population based study in the Madurai district of southern Tamil Nadu, India, estimated an annual incidence of corneal ulceration of 11.3 per 10,000 in the populations [2], which is ten times higher than the reported incidence from a study from the United States [3]. *Fusarium* and *Aspergillus*, are the predominant etiological agents responsible for 44% of all corneal ulcers [4]. Reports from Ghana [5] and northern Tanzania [6] also showed fungi as the etiological agent in over 50% of culture positive cases of keratitis. *Fusarium* species is more commonly associated with fungal keratitis in southern India, while *Aspergillus* species is more often implicated in northern India and Nepal [7]. The visual outcome following mycotic keratitis is generally poorer when compared to bacterial keratitis [8] and Natamycin, which is still the drug of choice for antifungal treatment is ineffective during late stages of the disease [9]. Therapeutic keratoplasty performed for mycotic keratitis is known to have a poorer prognosis [6], [10]–[11]. *Fusarium* sp., sometimes invade the anterior chamber and form a lens-iris-fungus mass at the pupillary area, thereby interfering with the normal drainage of the aqueous humor and leading to a rise in intraocular pressure [12], [13]. Predictive or diagnostic markers are not available to detect the preclinical infections, a stage that is suitable for effective treatment. Neither the molecular mechanism underlying the susceptibility of the host and virulence of the pathogen examined.

In this study we have examined the tear profile of patients with keratitis caused by *Fusarium.* The tear proteins play an important role in maintaining healthy ocular surface, and changes in tear protein components have been shown to reflect the changes in the health of the ocular surface [14]–[20]. Proteomics is a valuable approach to find patterns of protein markers for a given disease [21]. Gel based proteomic approach has been used to show the alterations induced at the post translational level in an infectious disease [22], [23]. Proteomic analysis can provide insights about protein expression patterns, which are associated with various pathological conditions and the identification of those tear proteins and their posttranslational modifications have the potential to reveal the mechanism of the disease [19], [24]–[28]. The best method available at present to examine lower-abundance proteins and to quantify expression changes with high confidence is fluorescence two-dimensional difference gel electrophoresis (2D-DIGE) [29]. This multiplex technology allows the detection and quantitation of differences between samples resolved on the same gel, or across multiple gels, when linked by an internal standard [30]. 2D-DIGE circumvents the main drawbacks associated with conventional bidimensional polyacrylamide gel electrophoresis (2D-PAGE), such as low sensitivity, reduced dynamic range, and gel-to-gel variability, enabling more accurate and sensitive quantitation of proteins [31]. We have shown earlier variation in the expression level of tear proteins (prolactin inducible protein, serum albumin precursor, cystatin S precursor, cystatin SN precursor, cystatin, and human tear lipocalin) in fungal keratitis patients [15].

The present study aims to identify quantitative changes in the keratitis tear protein obtained from *Fusarium* keratitis patients using 2D DIGE. We have used tear fluid from different stages of *Fusarium* infection. As far as we know, this is the first report showing quantitative proteome level analysis of tear fluid from mycotic keratitis patients. Further these changes are fungal infection specific.

## Results

### 2D DIGE analysis of Tear Proteins

**Figure 1:**
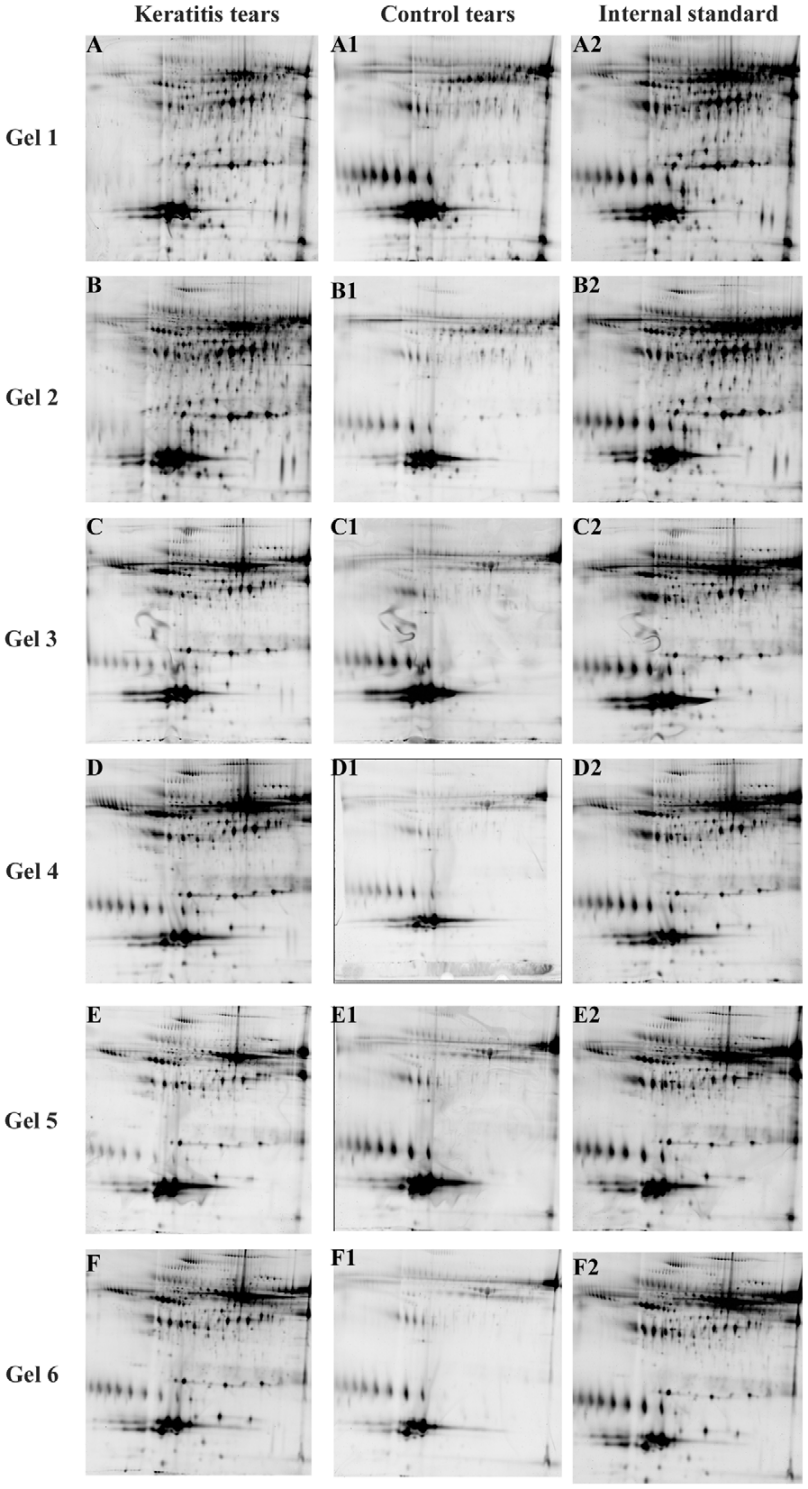
Fluorescent protein profiles of a set of six gels. Experimental design is described in Table 1. A&B represents *Fusarium* early keratitis; C&D represents *Fusarium* intermediate keratitis; E&F represents *Fusarium* late keratitis; A1–F1 represents control tears; A2–F2 represents the pooled internal standard. Each gel contained 90 µg of total protein separated by a pH 4–7 IPG strip in the first dimension and 12.5% polyacrylamide gel in the second dimension electrophoresis. Images were captured using a Typhoon Trio Variable Mode Imager.

The experimental design of 2D DIGE is described in Table 1, and eighteen images from six gels of a typical experiment are shown in Figure 1. The spot position in the fluorescent images generated by the Typhoon trio system was comparable to the silver or coomassie stained gels (data not shown).

**Table 1:** The DIGE Experimental Design for Minimal Labeling with Cy Dyes used in this study.

Gel No	Cy 3	Cy 5	Cy 2
1	*Fusarium* early (30 µg)	Control(30 µg)	Pooled Internal standard
2	Control(30 µg)	*Fusarium* early(30 µg)	Pooled Internal standard
3	*Fusarium* intermediate(30 µg)	Control(30 µg)	Pooled Internal standard
4	Control(30 µg)	*Fusarium* intermediate(30 µg)	Pooled Internal standard
5	*Fusarium* late(30 µg)	Control(30 µg)	Pooled Internal standard
6	Control(30 µg)	*Fusarium* late(30 µg)	Pooled Internal standard

A total of 90 µg of labeled proteins were loaded on each gel for 2D electrophoresis; Tear sample from early, intermediate and late stage keratitis patients were used. Labeling, construction of pooled standards are described under materials and methods. 250 pmoles of dye per 30 µg protein was used.

**Figure 2:**
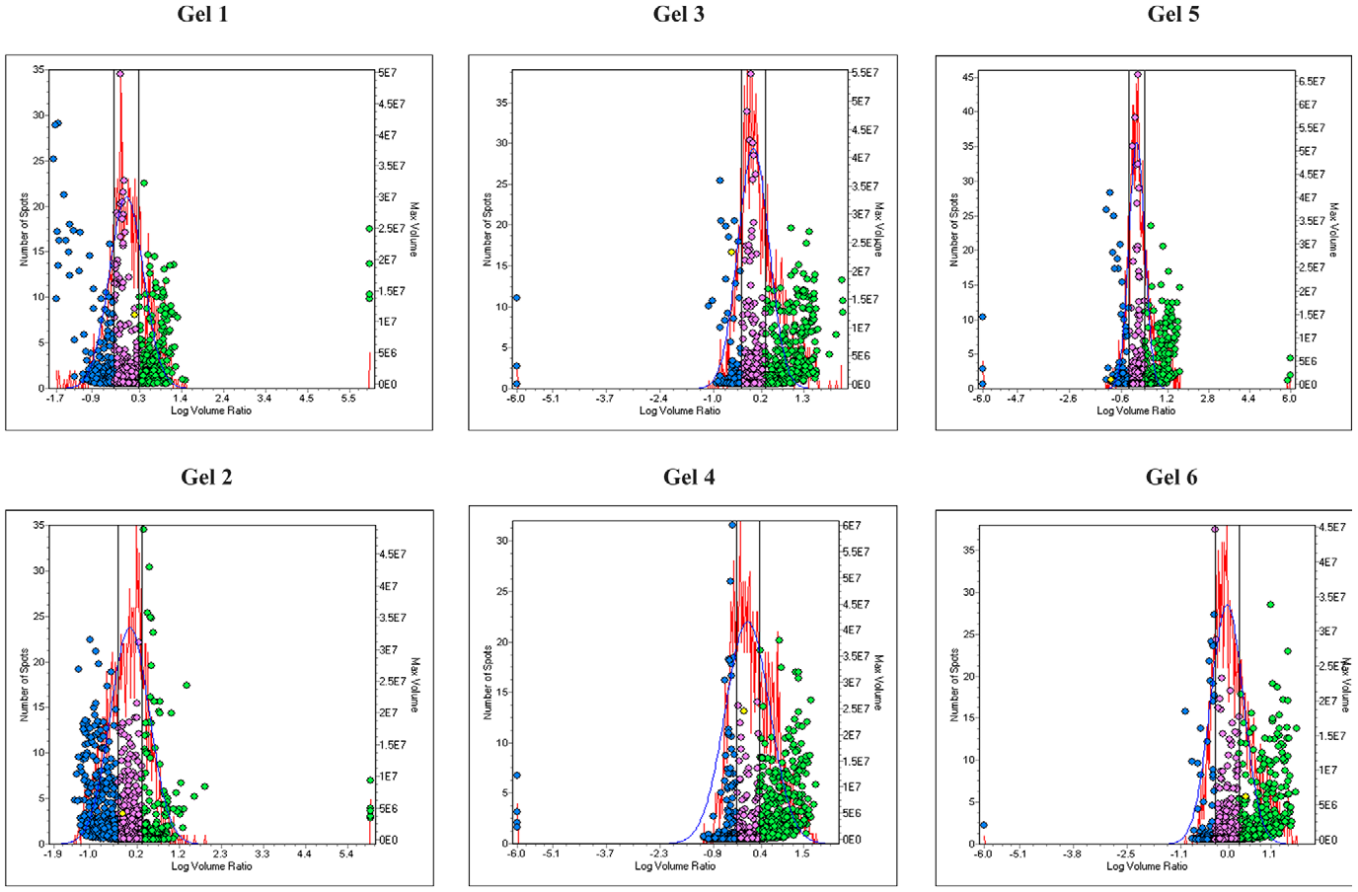
Discrimination of differentially expressed proteins in six gels. The histograms were generated by DeCyder DIA software, plotting spot frequency (left y-axis) against log volume ratio (x-axis), and a normal distribution model (red line) was fitted to the main peak of the frequency histogram. A 2 fold variation in expression level was set as threshold. The threshold values are represented by vertical black lines in the histogram, and the differentially expressed spot features are shown as blue spots (volume decreased) and green spots(volume increased). Table 2 describes the details of spots which show variable expression.

The protein co-detection of the three different CyDye labeled images in each gel was first performed by the DIA module of the software. To perform reliable quantification, there is a built-in normalization function (using the internal standard) to compensate for experimental variations, such as differences in laser power, fluorescence labeling, and sample loading. DIA module used in addition for quantitation of protein volume ratios between the co detected Cy3, Cy5 & Cy2 signals from a single gel. Cy2 forming the internal standard and the ratio of Cy3/Cy2 and Cy5/Cy2 were calculated in the DIA module. In this module variable of one (N = 1) is considered. In Figure 2 protein spots were plotted with the log volume ratio on the *x*-axis and the left *y*-axis shows the number of spots and the right *y*-axis shows the spot volume. Table 2 shows the DIA data of the spot pairs detected, and the total number of spots used in the calculation is also given. In Figure 2, the spots that show less than two fold variation are within the two vertical lines. Spots outside the vertical lines represent the spots that show more than two fold variation. The normalized spot volume data against internal standard allowed a rapid overview of the differential protein expressions.

**Table 2:** Details of spot detection and spot quantitation using DIA software.

GelNo	Cy3	Cy5	Spots detected	Max Volume Threshold	Decreased	Similar	Increased
1	*Fusarium* early	Control	1149	2	225(19.6%)	592(51.5%)	332(28.9%)
2	Control	*Fusarium* early	1455	2	316(21.7%)	682(46.9%)	457(31.4%)
3	*Fusarium* intermediate	Control	1578	2	253(16%)	824(52.2%)	501(31.7%)
4	Control	*Fusarium* intermediate	1533	2	350(22.8%)	641(41.8%)	542(35.4%)
5	*Fusarium* late	Control	1567	2	229(14.6%)	915(58.4%)	423(27%)
6	Control	*Fusarium* late	1525	2	242(51.9%)	792(51.9%)	491(32.2%)

DIA analysis was done as described in DeCyder software manual using default settings. Data for the threshold value of two alone is given. Dye swapping was performed to normalize variations in labeling.

### Comparison of quantitative changes in protein expression across gels

**Figure 3:**
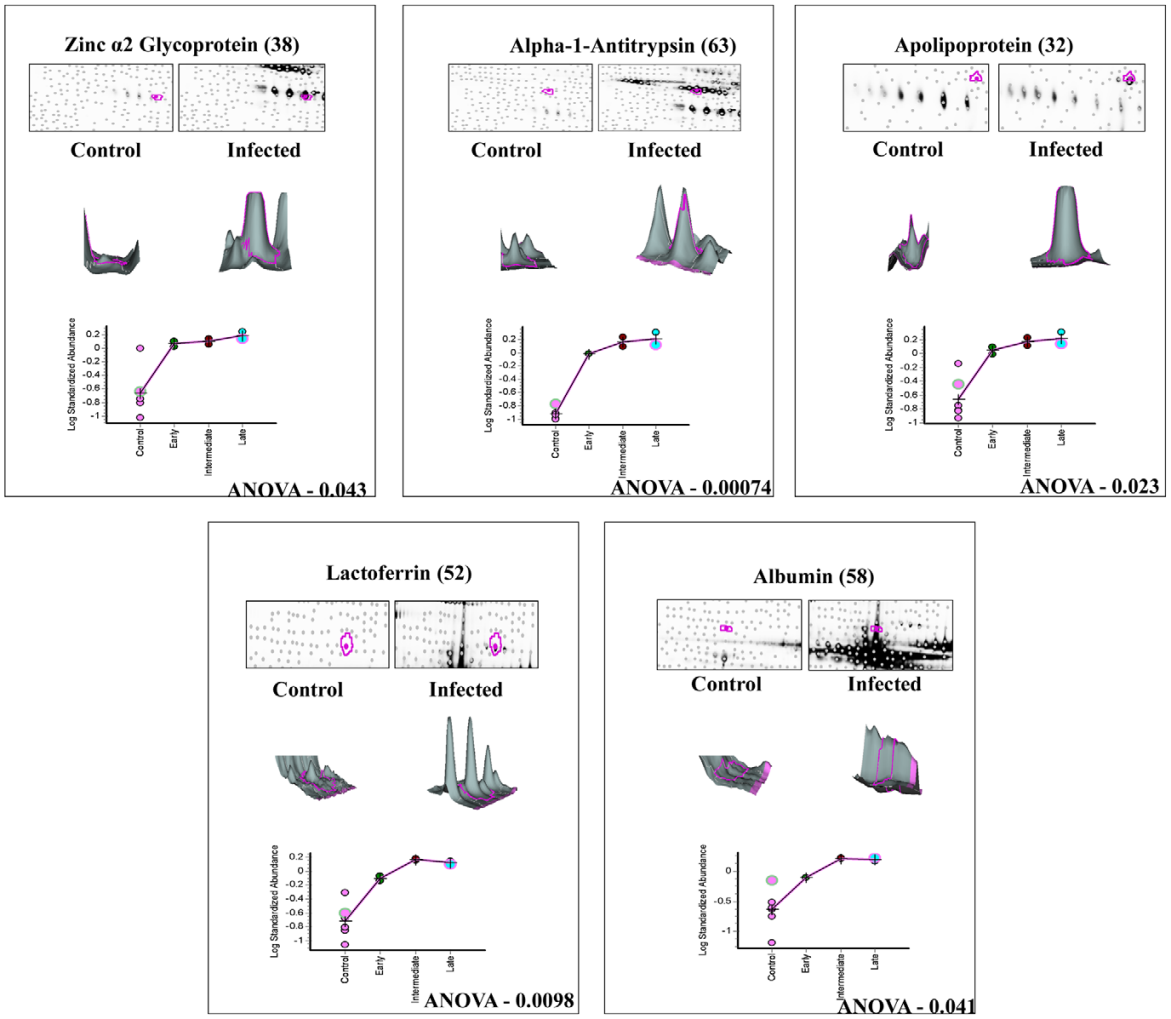
3D and graphical representation of expression of up regulated proteins. Graphic views show the standardized log abundance of spot volume (y-axis) against the changes of proteins between the control and infected groups (x-axis) in all six gels. 3-D view of control and late stage infection sample spots is also shown.

**Figure 4:**
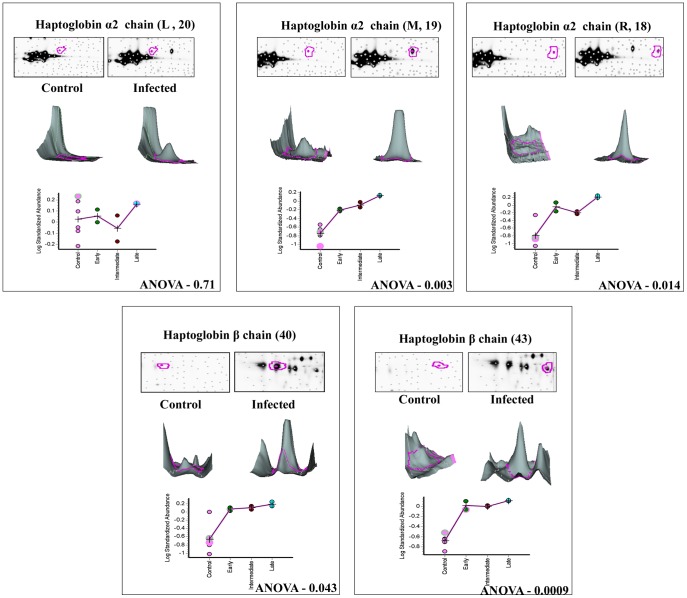
3D and graphical representation of expression of Haptoglobin α2 & β chain. Graphic views show the standardized log abundance of spot volume (y-axis) against the changes of proteins between the control and infected groups (x-axis) in all six gels. 3-D view of control and late stage infection sample spots is also shown.

**Figure 5:**
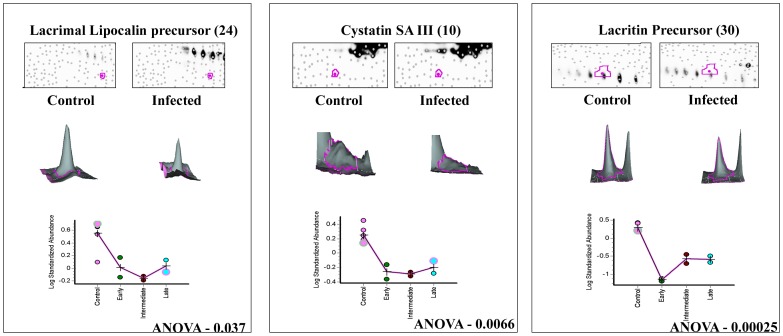
3D and graphical representation of expression of down regulated proteins. Graphic views show the standardized log abundance of spot volume (y-axis) against the changes of proteins between the control and infected groups (x-axis) in all six gels. 3-D view of control and late stage infection sample spots is also shown.

Differential protein expression of those co-detected pairs between the keratitis and control tear across all six gels representing different stages of infection were quantified using the BVA module. In order to avoid gel to gel variability, Isoelectric focusing and second dimension analysis were done using IPGphor III and Ettan Dalt six equipments, which allow simultaneous analysis of six samples. To detect variation in expression levels, relatively conservative selection criteria were used in image analysis. Differentially expressed proteins that fulfilled the following criteria alone were used for further analysis, 1) Minimum mean difference between the infected and control protein samples should be 1.5 fold or more, in terms of spot volume. 2) In all experiments an internal standard as described under materials and methods was included. 3) Built in t-test or one way ANOVA statistics software was used to detect significance of fold change (p≤0.05). 4) These spots were checked manually to confirm that they were true protein spots and not artifacts. More than 281 protein pairs (showed 1.5 fold variation or more in expression) were statistically significant (ANOVA p<0.05). However, upon application of the above stringent criteria only 140 protein pairs could be selected for further analysis. Among these 140 protein pairs, 116 spots showed higher expression level and 24 spots were down-regulated. Therefore, up-regulation of proteins seems to be the dominant features in the expression profiles after fungal infection. Similar analysis procedure was followed for all the gel comparisons and Table 3 shows the data. The data for ten differentially expressed proteins are shown in Figure 3, 4 and 5. Protein expression changes of a particular spot in each of the six gels in terms of the spot volume between the keratitis and control tears are shown. In addition, the 3-D representations of the spot volume are also shown. The BVA data was analyzed individually for Cy3 & Cy5 labeled gels (Table S1) to confirm the EDA data.

**Table 3:** BVA analysis of the data.

S.No	Description	Control Vs *Fusarium* (early, intermediate & late) keratitis tears	Control Vs *Fusarium* early keratitis tears	Control Vs *Fusarium* intermediate keratitis tears	Control Vs *Fusarium* late keratitis tears	*Fusarium* early Vs intermediate keratitis tears	*Fusarium* early Vs late keratitis tears	*Fusarium* intermediate Vs late keratitis tears
(A)	Total no. of paired spots detected	1578	1578	1578	1578	1578	1578	1578
(B)	Among (A) differentially expressed spots >1.5 fold difference in spot volume average ratio	1009	858	1129	854	750	551	496
(C)	Among (B) spots with ANOVA ≤0.05	281	243	279	261	163	124	90
(D)	Among (C) manually confirmed true spots	140	132	140	133	68	60	30
(E)	Among (D) Total number up regulated spots	116	109	116	110	54	49	12
(F)	Among (E) total number of spots identified	11	18	23	22	20	25	4
(G)	Among (D) Total number down regulated spots	24	23	24	23	14	11	18
(H)	Among (G) total number of spots identified	14	14	14	14	1	0	2

### Image Analysis by DeCyder Extended Data Analysis (EDA)

**Figure 6:**
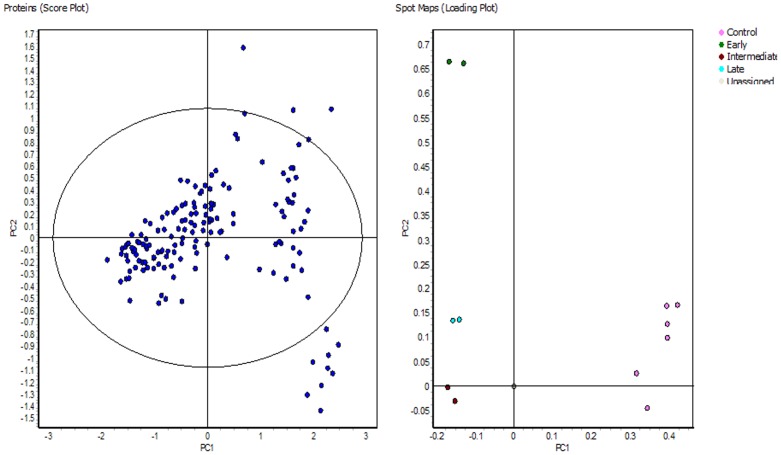
Clustering analysis of fusarium infected tear samples. Principal component analysis (PCA) has been performed across different experimental groups. A subset of those proteins whose expression varied within the 95th confidence level (ANOVA; *p*<0.01) was created. In this analysis the default settings of the software were used.

**Figure 7:**
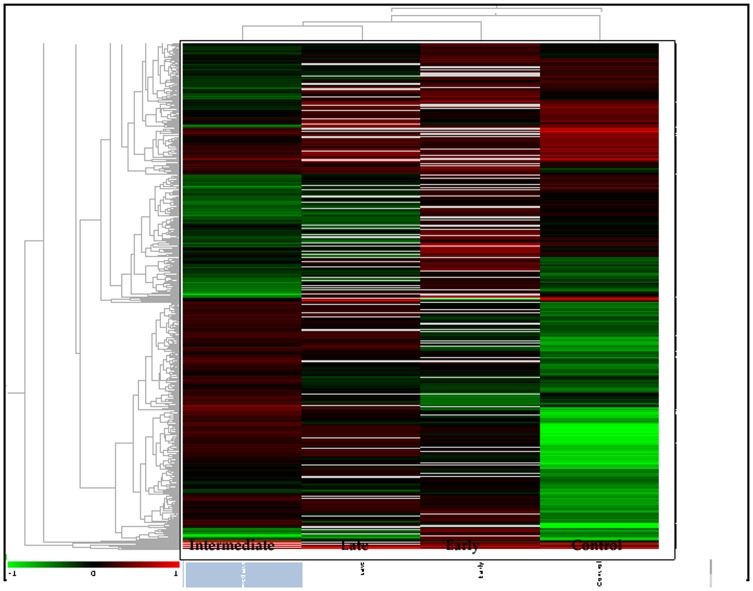
Unsupervised hierarchical of the expressed proteins. Log-transformed normalized protein spot volumes were used to perform unsupervised hierarchical cluster analysis. Green indicates decreased expression; red indicates increased expression. Patient groups; control and fusarium infected (Early, Intermediate and Late).

*Extended Data Analysis* (EDA) module was used for principal component analysis and clustering studies. Matched spots that showed significant (ANOVA p≤0.01) variation in their expression were transferred to the EDA module of the software. Default settings were used for intra and intergel statistical analyses allowing the characterization and classification of biological samples based on protein expression data. Principle component analysis (PCA), which is a statistical method to eliminate redundant variables and reduce data complexity, was also performed and the results are shown in Figure 6. An unsupervised clustering analysis was performed by selecting the hierarchical clustering algorithm available within the EDA software and Figure 7 shows the result. In all these analysis default settings of the software were used and hence the confidence of the data is assured.

### Dye swapping to minimize dye specific labeling efficiency

It has been reported that there was preferential lysine labeling of Cy2 to particular protein spot which has not been identified [32]. Since DIGE technology has not been applied extensively to tear sample, we have examined preferential labeling using a reciprocal labeling of the same protein. Therefore, with the reciprocal staining approach, any possible false positive results caused by the preferential labeling of dyes could be identified and corrected.

### Protein Identification by LC–MS/MS

**Figure 8:**
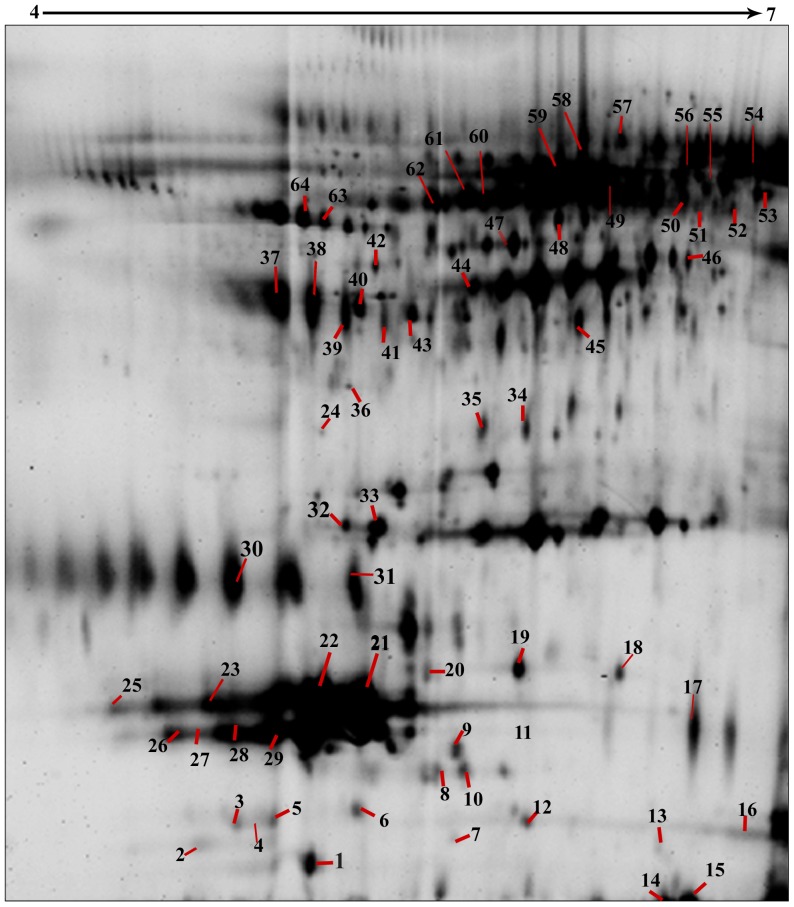
Tear protein profile showing the annotations of the identified tear proteins. 2D DIGE gel contained 30 µg of Cy2 labeled internal standard as described in Table 1 was separated by a pH 4–7 IPG strip in the first dimension and 12.5% polyacrylamide gel in the second dimension electrophoresis. Image was captured using a Typhoon Trio Variable Mode Imager.

Sixty four tear proteins were identified (Figure 8) using mass spectrometry as described under materials and methods. Some of the protein spots were isoforms and hence a total of 23 different proteins from 64 spots were identified. The details of the identified proteins are shown in Table S2. Among them ten proteins were found to have statistically significant variation in expression level. Cystatin SA III precursor (spot ID 10), lacrimal lipocalin precursor (spot ID 24) and lacritin precursor (spot ID 30) were down regulated, while alpha 1 antitrypsin (spot ID 63), apolipoprotein (spot ID 32), haptoglobin (spot ID 18 & 19), albumin (spot ID 58), zinc α 2 glycoprotein (ZAG) (spot ID 38), lactoferrin (spot ID 52), were up regulated in the keratitis tear compared to control tear (Table 4 & Figure 8).

**Table 4:** Identified tear proteins and their function.

Spot ID	Identified Tear proteins	Database/Accession no.	Mowse score^a^	X & Y coordinate in DIGE gel	M*r*/p*I* 2-D^b^	M*r*/p*I* data base^c^	Peptides Matched^d^	Sequence Coverage (%)^e^	Function
10	**cystatin SA-III = potential precursor of acquired enamel pellicle [human, Peptide, 121 aa]**	**NCBI/gi|235948**	**182(38)**	**484,835**	**16/5.4**	**14.1/4.7**	**R.IIPGGIYDADLNDEWVQR.A R.RPLQVLR.A K.SQPNLDTCAFHEQPELQK.K K.SQPNLDTCAFHEQPELQK.K**	**35**	**Cysteine protease inhibitor**
**19**	**Haptoglobin OS = Homo sapiens GN = HP PE = 1 SV = 1– α2 chain (M)**	**Swissprot/HPT_HUMAN**	**212 (35)**	**555,724**	**20/5.9**	**45.8/6.1**	**K.NYYKLR.T K.LRTEGDGVYTLNNEK.Q R.TEGDGVYTLNNEK.Q R.TEGDGVYTLNNEKQWINK.A**	**6**	**Haeme binding protein**
**24**	**Lacrimal lipocalin precursor**	**MSDB/LCHUL**	**112 (36)**	**174,733**	**19/4.4**	**19.2/5.39**	**K.NNLEALEDFEK.A R.GLSTESILIPR.Q**	**12**	**Lipid scavenging and transport to outer tear layer**
**30**	**Lacritin Precursor**	**NCBI/gi|15187164**	**62 (38)**	**244,622**	**26/4.8**	**14.2/5.43**	**K.SILLTEQALAK.A K.KFSLLKPWA**	**14**	**Secretion, renewal of lacrimal & ocular surface epithelia**
**32**	**Pro Apolipoprotein**	**NCBI/gi|178775**	**204 (27)**	**367,575**	**30/5.2**	**28.9/5.45**	**K.LLDNWDSVTSTFSK.L R.THLAPYSDELRQR.L K.ATEHLSTLSEK.A K.AKPALEDLR.Q**	**18**	**Lipid profile regulator**
**38**	**Zn-alpha2-glycoprotein [Homo sapiens]**	**NCBI/gi|38026**	**607 (36)**	**293,317**	**51/4.8**	**34.7/5.71**	**R.YSLTYIYTGLSK.H K.SQPMGLWR.Q R.QVEGMEDWK.Q R.QVEGMEDWKQDSQLQK.A K.AREDIFMETLK.D R.EDIFMETLK.D K.YYYDGKDYIEFNK.E K.QKWEAEPVYVQR.A K.WEAEPVYVQR.A K.AYLEEECPATLR.K K.AYLEEECPATLRK.Y R.QDPPSVVVTSHQAPGEK.K K.CLAYDFYPGK.I**	**37**	**Stimulates lipid degradation**
**40**	**Haptoglobin precursor – β chain**	**NCBI/gi|306882**	**182(35)**	**379,336**	**39/5.6**	**45.1/6.24**	**R.ILGGHLDAK.G K.GSFPWQAK.M K.DIAPTLTLYVGK.K K.QLVEIEK.V R.VGYVSGWGR.N K.FTDHLK.Y K.VTSIQDWVQK.T**	**15**	**Haeme binding protein**
**52**	**Lactoferrin**	**NCBI/gi|2104522**	**222 (38)**	**800,269**	**50/7**	**53.6/7.09**	**K.CGLVPVLAENYK.S R.SDTSLTWNSVK.G R.CLAENAGDVAFVK.D**	**9**	**Antimicrobial activity**
**58**	**Serum albumin, chain A**	**MSDB/1AO6A**	**639 (36)**	**550,131**	**66/5.1**	**65.6/5.63**	**R.FKDLGEENFK.A K.LVNEVTEFAK.T K.KYLYEIAR.R K.YLYEIAR.R K.VHTECCHGDLLECADDR.A K.VHTECCHGDLLECADDRADLAK.Y K.QNCELFEQLGEYK.F K.FQNALLVR.Y K.KVPQVSTPTLVEVSR.N K.VPQVSTPTLVEVSR.N K.QTALVELVK.H K.AVMDDFAAFVEK.C**	**18**	**Regulation of the colloidal osmotic pressure of blood**
**63**	**Alpha-1-antitrypsin**	**Swissprot/A1AT_HUMAN**	**76 (28)**	**269,210**	**45/5.2**	**46.8/5.3**	**K.TDTSHHDQDHPTFNK.I K.IVDLVK.E K.FLENEDRR.S K.LSITGTYDLK.S**	**9**	**Inhibitor of serine proteases**

a MOWSE scores greater than the values given in the parenthesis are considered to be significant (*p*<0.05). All proteins were also searched across multiple databases to confirm their identity.

b Apparent/experimental molecular weight and pI of protein spot on 2-DE gels.

c Theoretical molecular weight and p*I* of the identified protein in database.

d Represents the peptides matched.

e Sequence coverage (SC) represents the % aminoacid sequence covered in the protein by the matched peptides. 18R, 19M and 20L of α2 isoforms are labeled according to Gupta^22^ *et al.*, 2007.

### Gene ontology annotation of differentially expressed proteins

In order to better understand the biological function of differentially expressed proteins, gene annotation was performed using DAVID [33] 6.7 (a database for annotation, visualization and integrated discovery). Selected proteins were classified into three categories of cellular component, molecular function, and biological process (Table S3). All the proteins that showed altered expression levels were extracellular proteins. Proteins that have enzyme inhibitor activity (two were upregulated and two were downregulated) were the most common proteins in the category of molecular function. In the biological process category defense response proteins were most common and all of them were upregulated (n = 4).

### Differential regulation of tear proteins at different stages of infection

The patients with keratitis were classified as early, intermediate and late as described under materials and methods. Differential expression of the proteins were observed in all the three infective stages (Table 5) and progressive up regulation of the seven proteins was observed as the disease progressed towards late stages of infection. Expression of cystatin SA III precursor and lacrimal lipocalin precursor declined during the late stage of infection. Lacritin precursor level was reduced to negligible amounts in the early infective stage itself compared to control (Figure 5). DIGE analysis shows that haptoglobin β chain (spots 40 & 43; Figure 8) and α2 chains (spots 18, 19, & 20; Figure 8) were upregulated in infected tear samples. Further two isoforms of beta chain and all three isoforms of α2 chains could also be detected. However, based on database entry all these are classified as haptoglobin precursors by MS/MS search as was shown earlier [22].

**Table 5:** Differentially Expressed Proteins in Tears of keratitis patients.

Spot.ID	Protein Name	Gene ID	Pattern of regulation	Fold Variation	One way ANOVA p value	T – test p value
**63**	**Alpha-1-antitrypsin**	SERPINA1	**Up regulation**	**12.04**	**0.00074**	**2.8e-005**
**19**	**Haptoglobin α2 chain (M)**	HP	**Up regulation**	**4.93**	**0.0030**	**0.00050**
**38**	**Zinc-alpha-2-glycoprotein**	AZGP1	**Up regulation**	**4.37**	**0.043**	**0.0032**
**32**	**Apolipoprotein**	APOA2	**Up regulation**	**5.19**	**0.023**	**0.0014**
**58**	**Albumin**	ALB	**Up regulation**	**4.56**	**0.041**	**0.0059**
**40**	**Haptoglobin precursor - β chain**	HP	**Up regulation**	**10.96**	**0.0053**	**0.00024**
**52**	**Lactoferrin**	LTF	**Up regulation**	**5.35**	**0.0098**	**0.00088**
**24**	**Lacrimal lipocalin precursor**	LCN1	**Down regulation**	**−4.04**	**0.037**	**0.0038**
**10**	**Cystatin SA III Precursor**	CST4	**Down regulation**	**−3.22**	**0.0066**	**0.00029**
**30**	**Lacritin Precursor**	LACRT	**Down regulation**	**−9.64**	**0.00025**	**0.00036**

### Immunodetection of Lacritin, Lipocalin and Haptoglobin in Tear Fluid

**Figure 9:**
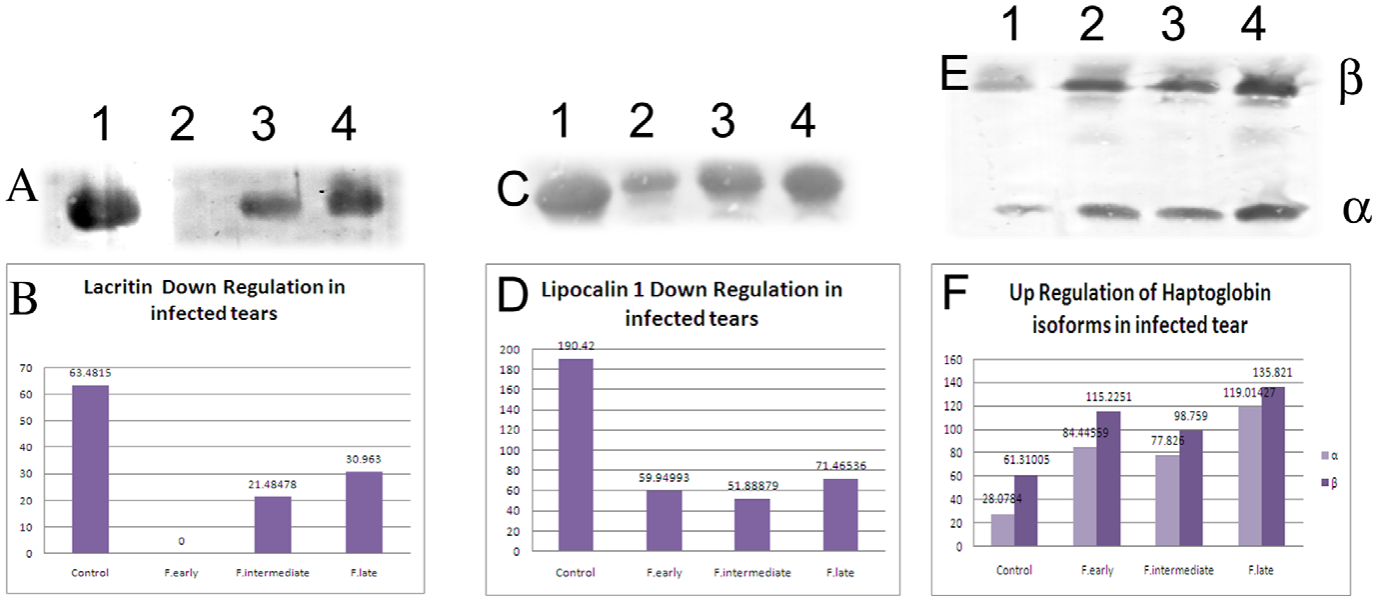
1-DE Immunoblot of keratitis patients tear proteins separated on 1D PAGE ; control subject (1) and tears of fusarium keratitis early stage (2), intermediate stage (3) and late stage (4) patients. Forty µg tear proteins were separated on 13.5% SDS PAGE. Tear proteins were transferred to nitrocellulose membranes for subsequent immunodetection with appropriate primary antibody. Secondary antibody used was HRP labeled and the images were scanned using a Typhoon Trio scanner as described under materials and methods. B, D and F are histograms showing the spot intensities.

**Figure 10:**
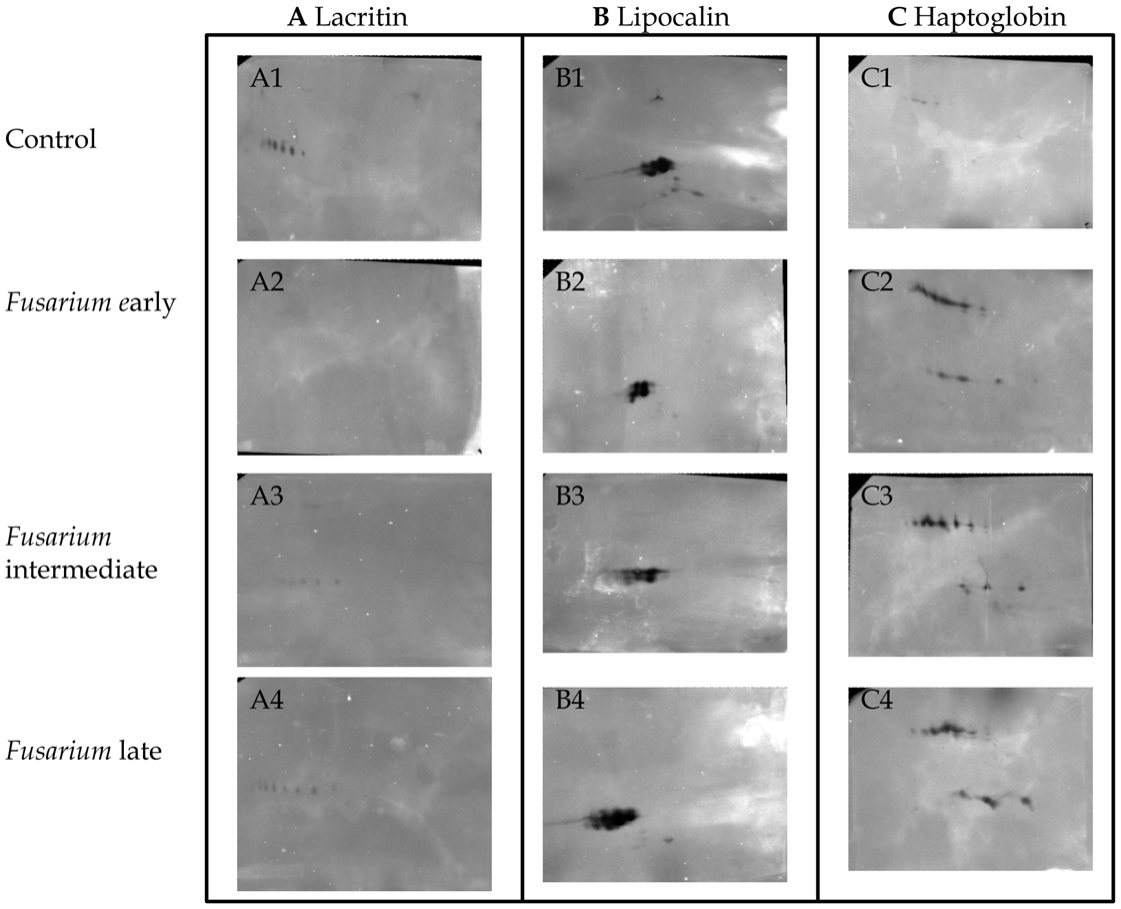
Immunoblot of tear proteins from control & keratitis patients separated on 2D PAGE. For 2-DE western blotting, 40 µg tear proteins was separated on pH 4–7 strip (7 cm), followed by second dimension separation on a 13.5% polyacrylamide gel. Tear proteins were transferred to nitrocellulose membranes for subsequent immunodetection with appropriate primary antibodies as described already.

Among the differentially regulated proteins, expression level of three proteins was confirmed using western blot analysis. 1D western blot analysis confirmed the reduction of lacritin and lipocalin levels and the increase of haptoglobin in tears from patients with *Fusarium* keratitis compared with control tears. The band volumes as determined using ImageQuantTL (GE Healthcare, Uppsala, Sweden) are shown in (Figure 9). 2D western analysis was performed to examine expression of isoforms. Western blot analysis confirmed that lacritin protein was totally absent in early stage of infection. Interestingly expression variation of lacritin and lipocalin isoforms in keratitis tears was also confirmed by western analysis (Figure 10). Upregulation of haptoglobin α and β chains in fungal keratitis tears was also confirmed using western blot analysis.

## Discussion

Tear proteins are derived from main and accessory lacrimal glands as well as proteins locally produced by corneal and conjunctival epithelial cells. Therefore tear is a useful sample to examine the expression of proteins in corneal infection.

One of the key steps that need careful attention in the tear proteomics is the method of collecting the tear. A recent study finds that there is no significant difference in the protein concentration in tears collected by capillary and schirmer collections, which are representatives of the reflex tear [34]. On loading the same amount of tear protein from normal and healthy control subjects, SDS PAGE reflected no significant change in the proteins as seen by the densitometry analysis. Most of the studies have used the capillary tear sample for the 2-D and MS analysis [35], [15]–[16], [28]. Capillary tear collection has been used for the 2-DE in disease conditions such as diabetic [20] and blepharitis patients [19]. Since the tear secretion is profusing, the capillary collection has been used in pathological conditions like infectious keratitis.

Another study [36] aimed to compare methods of tear film collection (i.e., capillary collection versus Schirmer collection). There were 84 proteins identified from protein associated with the Schirmer method and 43 identified from the capillary method. Only 30 total proteins identified overlapped between the two collection methods. The study proposes that this difference arises through the Schirmer strip's interaction with the epithelium of the ocular surface (whereas the capillary method does not). To help examine this hypothesis, analysis of the various classifications/functions of the proteins identified were grouped based on their general function as follows transport, metabolism, immune response, structure, antioxidation, protease inhibitors, unclassified, cell signaling, and protein folding. There are several cellular proteins (i.e., not secreted) observed from the Schirmer method that were not found in tear film collected by capillary such as the S100 calcium binding series of proteins. Interestingly, serum albumin was detected at much higher levels in the tears collected using the Schirmer collection method.

Four fold decrease of lacrimal lipocalin precursor was observed in the tears of patients with fungal keratitis. Lipocalin-1, is a major tear protein secreted by lacrimal glands and acts as the principal lipid binding protein and is involved in the general protection of epithelial cell surfaces. Tear lipocalin protein level in human tears ranges between 0.5 and 1.5 g/l, making up 10%–20% of the total tear protein content in tears and is considered tear-specific [37], [38]. Tissue specificity and concentration reflects the importance of lipocalin in the protection of the ocular surface [39]. Based on their possible role in lipid transport [40] lipocalin were called as tear-specific prealbumin [41].

Cystatins are cysteine proteinase inhibitors belonging to the cystatin superfamily. The role of cystatins as proteinase inhibitors is well established. The balance between proteases secreted by the pathogen and protease inhibitor is believed to play a significant role in maintaining the normal ocular surface condition [42]. Human salivary cystatins are represented as Cystatin S. It contains three molecular species (cystatin S, cystatin SN and cystatin SA), which have distinct amino-acid sequences. Cystatin S binds cysteine proteases and inhibit their activity, thereby preventing uncontrolled proteolysis and tissue damages [43]–[46]. Cystatin S precursor was shown to be upregulated in healthy female tears compared to healthy male tears [47]. In tears of patients with blepharitis, significantly lower levels of cystatins SA have been reported [19]. The three fold reduction of cystatins in the tears of patients with fungal keratitis correlates well with their functional role. Cysteine protease inhibitors are reduced in several pathological conditions [48].

Ten fold reduction in the level of lacritin in the infected group compared to control may have relevance to resistance to fungal infection. Lacritin is a glycoprotein found in the tears that is primarily produced by the lacrimal gland, but has also been shown to be produced by the meibomian glands, corneal and conjunctival epithelia. The protein is an extracellular growth factor thought to be associated with cell proliferation as a secretory mitogen [49], [50]. It is a new lacrimal functional unit specific growth factor in human tears and flows through the ducts to target corneal epithelial cells on the ocular surface. This protein enhances unstimulated secretion and rapid tyrosine phosphorylation in lacrimal acinar cells, and it also stimulates corneal epithelial cell calcium signaling. Therefore, lacritin may play a key role in the function of the lacrimal gland-corneal axis and its deficiency could lead to cause some alterations in the defense mechanism. Lacritin might be important for tear secretion and maintaining normal tear conditions. Lacritin has been shown to be decreased in conditions altering tear film such as blepharitis [19] and dry eye, indicating the crucial role of this protein in infection and other conditions [34], [51]–[52]. Reductions in the level of lipocalin precursor and lacritin have direct effect in the pathogenesis and susceptibility of host corneal tissue. Inhibition of the protease inhibitor cystatins may lead to better survival of the pathogen.

Alpha-1 antitrypsin is an enzyme that controls the activity of diverse proteolytic enzymes such as trypsin, chymotrypsin, collagenase, thrombin, fibrinolysin, granulocytic proteases and caseins by cleaving their catalytic sites [53]. This protein is found in a number of body fluids such as tears, perilymph, lymph, saliva, colostrum, breast milk, duodenal fluid, gallbladder bile, synovial fluid, cervical mucus, semen and amniotic fluid [54]–[57] and plays an important role in both physiological and pathological condition by inactivating enzymes activated by bacteria or other agents. This protein also regulates [58] the immune response by inhibiting the transformation and migration of lymphocytes. The increased level of tear alpha-1 antitrypsin in corneal ulcer and other diseases have been reported [59], [60]. It has been shown that tear alpha-1 antitrypsin levels may increase in inflamed eyes in the absence of corneal involvement, for example, in cases of rosacea keratitis and allergic conjunctivitis [61], [62]. All these studies imply that alpha-1-antitrypsin might be an acute-phase reactant [63]. In fungal keratitis, the increase in alpha-1-antitrypsin could be due to the inflammation induced by the pathogen or a specific pathogen component may be involved.

Haptoglobin (Hp) is a plasma glycoprotein, the main biological function of which is to bind free hemoglobin (Hb). It is also a positive acute-phase protein with immunomodulatory properties [64]–[66]. Increase in tear haptoglobin alpha levels has been shown in conjunctivitis [65]. In our study haptoglobin β chain isoforms and α2 chain isoforms were upregulated in infected tear samples but not in control tear. The fungal infection presumably leads to the increased synthesis and export into tear samples. Since both α & β forms are found, it is interesting to examine whether functional α2β complexes are formed in the tear.

Zinc α2-glycoprotein (ZAG) is a 40-kDa single-chain polypeptide, which is secreted in various body fluids such as plasma, saliva, and tears [67]. It stimulates lipid breakdown in adipocytes [68] and is associated with the extreme weight loss that occurs in some cancers [69]. ZAG is shown to be increased in patients with Graves's ophthalmopathy [70]. The exact function of ZAG in tears and its possible role in lipid degradation are not demonstrated [70]. In our study, we detected significant upregulation of ZAG in keratitis patients, implying the alteration in the lipidome profile of tear in infection.

Apolipoproteins are proteins that bind to lipids (oil-soluble substances such as fat and cholesterol) to form lipoproteins, which transport the lipids through the lymphatic and circulatory systems. Apolipoproteins also serve as enzyme cofactors, receptor ligands and lipid transfer carriers that regulate the metabolism of lipoproteins and their uptake in tissues. Increased secretion of native apo A-I from the main lacrimal gland in patients with advanced diabetic retinopathy has been reported [71]. Apolipoprotein level was increased fivefold in all the three stages of fungal keratitis tears compared to control tears.

The mean albumin level in serum was reported as 44.7 mg/ml in the healthy individuals from India [72]. Albumin levels in tear fluids were reported to be 11.5 mg/l in healthy males and 12.7 mg/l in healthy females from Indian population [60]. In cases of allergic conjunctivitis tear levels of serum albumin reach serum levels rapidly, indicating a direct leak from inflamed vessels. The albumin content of tears also increases in cases of corneal ulceration [59]. Our earlier report [15] and our present results show albumin upregulation in keratitis tear sample indicating that during fungal infection there might be a leakage of this protein from inflamed vessels.

Tear lactoferrin (formerly known as lactotransferrin) is an 82 kDa protein produced by the acinar cells of the lacrimal gland. Lactoferrin is present in normal tears of humans and its level was found to be invariant through age. Lactoferrin is a glycoprotein, and a member of the transferrin family, belong to those proteins capable of binding and transferring Fe_3+_ ions [73]. Due to the increase in its concentration during most inflammatory reactions and some viral infections, several reports classify lactoferrin as an acute-phase protein [74]. Its concentration increases in all biological fluids, but the highest levels have been detected in the nidus of inflammation [75]. Lactoferrin has a wide variety of biological functions, many of which do not appear to be connected with its iron binding ability [76]. Its role in fungal infection is not clear as of now.

### Functional Implication of Differentially Expressed Tear Proteins at different infective stages

As the infective stage progressed seven acute phase proteins were upregulated. Interestingly 2D western analysis reveals lipocalin and lacritin isoforms are at a negligible level in the early stage of fungal infection, but their level increase marginally during intermediate stage. This increase could be due to the fungal elaboration and the induction of inflammatory response. Taken together these observations imply that the treatment options needs to be tailored based on the stage of infection.

2D-DIGE analysis of tears from fungal keratitis patients allowed the confident quantification of several differentially expressed proteins in comparison to normal tears. Fungal infection of cornea clearly induce increased expression of alpha 1 antitrypsin, apolipoprotein, haptoglobin, albumin, zinc α 2 glycoprotein (ZAG), lactoferrin, haptoglobin precursor, proteins related to inflammatory events. Expression of proteins related to host immune response such as cystatin SA III potential precursor, lacrimal lipocalin precursor, lacritin precursor is reduced in infection. Quantitation of lacritin levels showed that this protein is down regulated significantly in fungal keratitis when compared to bacterial keratitis tears (data not shown). This information and other results (unpublished) show that the changes reported in this paper are specific to fungal keratitis.

Additional studies focusing on the functional properties of these proteins in the pathogenesis of fungal keratitis may contribute to a better understanding of the disease. In addition these proteins form putative candidate biomarkers that could aid in the diagnosis, prognosis and treatment of fungal keratitis.

## Materials and Methods

### Tear Samples

Written Informed consents were obtained from study subjects and the study was approved by the Institutional Review Board of Aravind Medical Research Foundation (Madurai, India). The Declaration of Helsinki was adhered to when enrolling subjects. Reflex tear samples (100–150 µl) were collected from the keratitis eye of culture positive (*Fusarium*) patients who were categorized based on the duration of the infection as early (within 7 days), intermediate (7–14 days) and late (after 14 days) stage of infection. Tears from healthy individuals (whose corneas were clear and devoid of any infection & inflammation) served as controls (60–80 µl). All the tear samples were obtained from male individuals with the age range from 20–60 yrs. The total number of tear samples collected for each category of presentation is as follows, early –35 ; intermediate – 20 ; late – 11. Among them five samples from each category were included for this study. Tear samples were collected using 10 µl-capillary tubes without touching the eye globe or the lid. The samples were centrifuged at 7800× g for 10 min at 4°C to remove cellular debris and stored in liquid nitrogen until analysis. Samples were collected from patients before treatment. Protein concentration was estimated using Bradford's method [77]. Samples used in this study were described in table S4.

### Two-dimensional difference gel electrophoresis

#### Sample preparation

Tears samples from *Fusarium* keratitis patients were pooled (250 µg of protein per patient). Three pools each were made from early, intermediate and late stage infection samples. Each pool was made from tear from five patients. Tear samples from 20 healthy individuals were pooled and used as control samples (100 µg of protein per healthy individual). Pooled tear samples were concentrated using 3 kDa Amicon Ultra centrifugal device (MILLIPORE, County Cork, Ireland) as per manufactures instructions with minor modifications. Briefly, tear sample (2000–2500 µg) was diluted to 600 µl with MilliQ water and placed into a 3 kDa Amicon Ultra centrifugal device and centrifuged at 13,000× g for 10 min; under these conditions the volume of the concentrate was reduced to 100 µl. An additional 500 µl of MilliQ water was added and the mixture was centrifuged as above.

### Proteins Labeling with CyDyes

Lysine labeling protocol, (minimal labeling) was used in this study. Each dye (2 nmol) was reconstituted with 5 µL anhydrous *N*,*N*-dimethylformamide (Sigma Aldrich chemical co.) and 400 pmol/µl stock dye was stored at −20°C. The stock dye was further diluted to 100 pmol/µl and this working dye solution was used immediately. The processed tear proteins were then labeled individually with dyes Cy3 and Cy5 while the pooled tear proteins prepared by mixing equal aliquot of protein from all samples in an experimental set up were labeled with Cy2 and was used as internal standard. A 30 µg portion of each group was mixed with 2.5 µL of dye and incubated on ice for 30 min in the dark. In the case of Cy2 labeling, total mix was labeled and 30 µg aliquot was used in each gel. The labeling reactions were quenched with 2 µL of 10 mM lysine and incubation was continued on ice for 10 min. Cy3 & Cy5 labeled samples were mixed with the Cy2-labeled samples, and an equal volume of lysis buffer containing 100 mM DTT and 2% (v/v) IPG buffer was added and incubated at room temperature for 10 min. The final volume for all preparations was adjusted to a total of 340 µL with rehydration buffer (7 M urea, 2 M thiourea, 1% IPG buffer, 50 mM DTT, 4% CHAPS, and a trace amount of bromophenol blue). A reciprocal labeling experiment was also performed. The labeling protocols for the 2D DIGE experiments are shown in Table 1.

### Two dimensional gel electrophoresis

Two dimensional gel electrophoresis of Cy dye labeled proteins was done as described already [78] with the following modifications. Eighteen cm IPG strips of pH 4–7 (GE Healthcare, Uppsala, Sweden) were employed in the first dimension. Labeled proteins were focused for a total of 80,000 Vhs at a constant temperature (20°C) under linear voltage ramp after an active IPG rehydration at 30 V in a IPGPhor III (GE Healthcare, Uppsala, Sweden) apparatus with following IEF conditions 500 V step-n-hold for 1 h, 1000 V gradient for 1 h, 8000 V gradient for 3 h and 8000 V step-n-hold for 8 h. Following IEF, each IPG strip was placed in the equilibration buffer containing 2% DTT first followed by incubation in another buffer in which the DTT was replaced by 2.5% iodoacetamide. The second dimension PAGE (12.5%) was carried out in an Ettan DaltSix systems (GE Healthcare, Uppsala, Sweden) at 1 W/gel for 1 hr and 13 W/gel for 5 hr. All experimental procedures were performed in dim light or in the dark.

### Protein Visualisation and DeCyder image analysis

After second dimension electrophoresis, the gels were scanned with Typhoon TRIO Variable Mode Imager (GE Healthcare, Uppsala, Sweden). All laser power adjustments and image acquisition were done using default settings of the Typhoon Scanner Control program. Cy2, Cy3 & Cy5 images were captured using the settings recommended by the manufacturer. The scanned gels were saved as modified 16-bit.gel format which gave good gray-scale representation covering the dynamic range of protein spots. Prior to analysis, all gel images were cropped to identical size in ImageLoader module of DeCyder version 7.0 (GE Healthcare, Uppsala, Sweden) and then transferred to the DeCyder Differential Analysis Software,version 7.0. The standardized spot volume (the sum of pixel values within a detected spot boundary) was calculated as the ratio between the absolute volume of the individual spot and the total volume of all spots in the gel. The internal standard (Cy2) on each gel intrinsically linked all gel images (Cy3 and Cy5) within the same gels and across all the gels in the specific set of an experiment. A DeCyder differential in-gel analysis (DIA) module was used for image analysis between samples within the same gel while a DeCyder biological variation analysis (BVA) module was performed for pairwise image analysis among multiple gels. Ratios of differentially expressed proteins were shown as “fold” changes between the spots of keratitis tears and the control tears. An increase of protein abundance in the keratitis tear was expressed as a positive value while a negative value denoted a decrease in the protein abundance. Protein spot has been normalized using the corresponding spot on the pooled internal standard on every gel. Student's *t*-test and ANOVA were used to compare the average spot volume and differences of protein abundance for all detectable spots between the keratitis and control groups. Reciprocal dye labeling was performed to normalize bias in labeling.

### Protein Identification and Mass Spectrometry

Pooled proteins (300 µg) from control tear group were separated on 18 cm IPG strips of pH 4–7 in the first dimension. First and second dimension electrophoresis were done as given under 2D DIGE method. The second dimension gels were stained with colloidal coomassie blue G-250 [79] and gel spots from this preparative gel were excised manually for in-gel trypsin digestion [15], and LC-MS/MS was performed. The resulting peptides were separated by an electrospray ionisation (ESI) quadrupole time-of-flight (Q-TOF) Bruker MicrOTOF Q (Bruker Daltonics) coupled with a nano liquid chromatography system (Ultimate 3000, Dionex, Hongkong). After data acquisition, the generated XML files were used to perform database searches using the MASCOT software v. 2.2 (Matrix Science, London, UK) using the following parameters: peptide tolerance, 0.2 Da; MS/MS ion mass tolerance, 0.1 Da; one missed cleavage; variable modifications used were methionine oxidation and cysteine carboxyamidomethylation.

### Gene Ontology by DAVID software

Analysis of functional enrichment of differentially regulated proteins was performed in DAVID 6.7 software [34] http://david.abcc.ncifcrf.gov/ according to the standard protocol [80]. The combined list of official gene symbols corresponding to the identified proteins was used for input.

### 1-D and 2-D Western Blot Analysis

Proteins (40 µg) were resolved either by SDS-PAGE or 2-DE and transferred onto a nitrocellulose membrane (GE Healthcare, Uppsala, Sweden) by semidry blotting using a Trans-Blot SD Semi-Dry Electrophoretic Transfer Cell (Bio-Rad Laboratories). Membrane was presoaked and equilibrated in Towbin transfer buffer [25 mM Tris, 192 mM glycine (20% methanol)]. Nonspecific binding was blocked with 5% skim milk in phosphate buffered saline (PBS). Primary antibodies used for immunodetection were given in Figure legends. They were obtained from the following sources Dako Denmark A/S, Denmark; Santa Cruz,USA; kindly provided by Robert McKown, James Madison University and Gordon Laurie, University of Virginia.

Immunodetection was performed by incubating the membranes in antibody solutions; The list of antibodies used include the following- Polyclonal rabbit anti-human haptoglobin (Dako Denmark A/S, Denmark), polyclonal rabbit anti lipocalin 1 (Santa Cruz,USA) and polyclonal rabbit anti lacritin (kindly provided by Robert McKown, James Madison University and Gordon Laurie, University of Virginia). The antibodies were diluted in 1% BSA prepared in Tween PBS (TPBS) and used for incubation with the membrane at RT for 2 h. After three washes with 0.5% TPBS, the membrane was exposed to secondary antibody conjugated with horseradish peroxidase Goat anti Rabbit Ig G (Jackson ImmunoResearch Laboratories, USA) diluted 1∶2000 in 0.5% TPBS for one hour at RT. The membranes were then incubated with 4Chloro-Naphthol substrate for 30 minutes. The membranes were rinsed with water thrice and documented using Typhoon Trio variable mode scanner (with TAMRA, Alexaflour 546) (GE Healthcare, Uppsala, Sweden) and Image analysis was done using ImageQuantTL software (GE Healthcare, Uppsala, Sweden) was used for image analysis.

## Supporting Information

Table S1 Differentially Expressed Proteins in Tears of keratitis patients.
(DOCX)

Table S2 Identified Tear proteins and their functions.
(DOCX)

Table S3 Classification of differentially regulated proteins according to biological process, cellular & molecular.
(DOCX)

Table S4 Tear sample details used in this study.
(DOCX)

## References

[1] Whitcher JP, Srinivasan M (1997) Corneal ulceration in the developing world-a silent epidemic. The British Journal of Ophthalmology 81: 622–623. 9349145 10.1136/bjo.81.8.622 PMC1722289

[2] Gonzales CA, Srinivasan M, Whitcher JP, Smolin G (1996) Incidence of corneal ulceration in Madurai district, South India. Ophthalmic Epidemiology 3: 159–166. 8956320 10.3109/09286589609080122

[3] Erie JC, Nevitt MP, Hodge DO, Ballard DJ (1993) Incidence of ulcerative keratitis in a defined population from 1950 through 1988. Archives of Ophthalmology 111: 1665–1671. 8155038 10.1001/archopht.1993.01090120087027

[4] Srinivasan M, Gonzales CA, George C, Cevallos V, Mascarenhas JM, et al (1997) Epidemiology and aetiological diagnosis of corneal ulceration in Madurai, south India. The British Journal of Ophthalmology 81: 965–971. 9505820 10.1136/bjo.81.11.965 PMC1722056

[5] Hagan M, Wright E, Newman M, Dolin P, Johnson G (1995) Causes of suppurative keratitis in Ghana. The British Journal of Ophthalmology 79: 1024–1028. 8534648 10.1136/bjo.79.11.1024 PMC505322

[6] Poole TRG, Hunter DL, Maliwa EMK, Ramsay ARC (2002) Aetiology of microbial keratitis in northern Tanzania. The British Journal of Ophthalmology 86: 941–942. 12140229 10.1136/bjo.86.8.941 PMC1771226

[7] Upadhyay MP, Karmacharya PC, Koirala S, Tuladhar NR, Bryan LE, et al (1991) Epidemiologic characteristics, predisposing factors, and etiologic diagnosis of corneal ulceration in Nepal. American Journal of Ophthalmology 111: 92–99. 1985498 10.1016/s0002-9394(14)76903-x

[8] Thomas PA, Geraldine P (2007) Infectious keratitis. Current Opinion in Infectious Diseases 20: 129–141. 17496570 10.1097/QCO.0b013e328017f878

[9] Srinivasan M (2004) Fungal keratitis. Current Opinion in Ophthalmology 15: 321–327. 15232472 10.1097/00055735-200408000-00008

[10] Levin LA, Avery R, Shore JW, Woog JJ, Baker AS (1996) The spectrum of orbital aspergillosis: a clinicopathological review. Survey of Ophthalmology 41: 142–154. 8890440 10.1016/s0039-6257(96)80004-x

[11] Yohai RA, Bullock JD, Aziz AA, Markert RJ (1994) Survival factors in rhino-orbital-cerebral mucormycosis. Survey of Ophthalmology 39: 3–22. 7974189 10.1016/s0039-6257(05)80041-4

[12] Jones BR (1975) Principles in the management of oculomycosis. XXXI Edward Jackson memorial lecture. American Journal of Ophthalmology 79: 719–75. 1096622 10.1016/0002-9394(75)90730-8

[13] Kuriakose T, Thomas PA (1991) Keratomycotic malignant glaucoma. Indian Journal of Ophthalmology 39: 118–121. 1841884

[14] Vasanthi M, Prajna NV, Lalitha P, Mahadevan K, Muthukkaruppan V (2007) A pilot study on the infiltrating cells and cytokine levels in the tear of fungal keratitis patients. Indian Journal of Ophthalmology 55: 27–31. 17189883 10.4103/0301-4738.29491

[15] Ananthi S, Chitra T, Bini R, Prajna NV, Lalitha P, et al (2008) Comparative analysis of the tear protein profile in mycotic keratitis patients. Molecular Vision 14: 500–507. 18385783 PMC2268856

[16] de Souza GA, Godoy LMF, Mann M (2006) Identification of 491 proteins in the tear fluid proteome reveals a large number of proteases and protease inhibitors. Genome Biology 7: R72. 16901338 10.1186/gb-2006-7-8-r72 PMC1779605

[17] Zhou L, Beuerman RW, Foo Y, Liu S, Ang LPK, Tan DTH (2006) Characterisation of human tear proteins using high-resolution mass spectrometry. Annals of the Academy of Medicine, Singapore 35: 400–407. 16865190

[18] Li N, Wang N, Zheng J, Liu XM, Lever OW, et al (2005) Characterization of human tear proteome using multiple proteomic analysis techniques. Journal of Proteome Research 4: 2052–2061. 16335950 10.1021/pr0501970

[19] Koo BS, Lee DY, Ha HS, Kim JC, Kim CW (2005) Comparative analysis of the tear protein expression in blepharitis patients using two-dimensional electrophoresis. Journal of Proteome Research 4: 719–724. 15952718 10.1021/pr0498133

[20] Herber S, Grus FH, Sabuncuo P, Augustin AJ (2001) Two-dimensional analysis of tear protein patterns of diabetic patients. Electrophoresis 22: 1838–1844. 11425240 10.1002/1522-2683(200105)22:9<1838::AID-ELPS1838>3.0.CO;2-7

[21] Righetti PG, Castagna A, Antonucci F, Piubelli C, Cecconi D, et al (2005) Proteome analysis in the clinical chemistry laboratory: myth or reality? Clinica Chimica Acta; International Journal of Clinical Chemistry 357: 123–139. 15970281 10.1016/j.cccn.2005.03.018

[22] Gupta N, Shankernarayan NP, Dharmalingam K (2007) Serum proteome of leprosy patients undergoing erythema nodosum leprosum reaction: regulation of expression of the isoforms of haptoglobin. Journal of Proteome Research 6: 3669–3679. 17658739 10.1021/pr070223p

[23] Gupta N, Shankernarayan NP, Dharmalingam K (2010) Alpha1-acid glycoprotein as a putative biomarker for monitoring the development of the type II reactional stage of leprosy. Journal of Medical Microbiology 59: 400–407. 20075114 10.1099/jmm.0.016394-0

[24] Zolg W (2006) The proteomic search for diagnostic biomarkers: lost in translation? Molecular & Cellular Proteomics 5: 1720–1726. 16546995 10.1074/mcp.R600001-MCP200

[25] Lescuyer P, Hochstrasser D, Rabilloud T (2007) How shall we use the proteomics toolbox for biomarker discovery? Journal of Proteome Research 6: 3371–3376. 17655344 10.1021/pr0702060

[26] Good DM, Thongboonkerd V, Novak J, Bascands JL, Schanstra JP, et al (2007) Body fluid proteomics for biomarker discovery: lessons from the past hold the key to success in the future. Journal of Proteome Research 6: 4549–4555. 17970587 10.1021/pr070529w

[27] Tomosugi N, Kitagawa K, Takahashi N, Sugai S, Ishikawa I (2005) Diagnostic potential of tear proteomic patterns in Sjogren's syndrome. Journal of Proteome Research 4: 820–825. 15952728 10.1021/pr0497576

[28] Molloy MP, Bolis S, Herbert BR, Ou K, Tyler MI, et al (1997) Establishment of the human reflex tear two-dimensional polyacrylamide gel electrophoresis reference map: new proteins of potential diagnostic value. Electrophoresis 18: 2811–2815. 9504814 10.1002/elps.1150181516

[29] Unlu M, Morgan ME, Minden JS (1997) Difference gel electrophoresis: a single gel method for detecting changes in protein extracts. Electrophoresis 18: 2071–2077. 9420172 10.1002/elps.1150181133

[30] Alban A, David SO, Bjorkesten L, Andersson C, Sloge E, et al (2003) A novel experimental design for comparative two-dimensional gel analysis: two-dimensional difference gel electrophoresis incorporating a pooled internal standard. Proteomics 3: 36–44. 12548632 10.1002/pmic.200390006

[31] Marouga R, David S, Hawkins E (2005) The development of the DIGE system: 2D fluorescence difference gel analysis technology. Analytical and Bioanalytical Chemistry 382: 669–678. 15900442 10.1007/s00216-005-3126-3

[32] Tonge R, Shaw J, Middleton B, Rowlinson R, Rayner S, et al (2001) Validation and development of fluorescence two-dimensional differential gel electrophoresis proteomics technology. Proteomics 1: 377–396. 11680884 10.1002/1615-9861(200103)1:3<377::AID-PROT377>3.0.CO;2-6

[33] Dennis G Jr, Sherman BT, Hosack DA, Yang J, Gao W, et al (2003) DAVID: Database for Annotation, Visualization, and Integrated Discovery. Genome Biology 4: P3. 12734009 PMC193660

[34] Saijyothi AV, Angayarkanni N, Syama C, Utpal T, Shweta A, et al (2010) Two dimensional electrophoretic analysis of human tears: collection method in dry eye syndrome. Electrophoresis 31: 3420–3427. 20882555 10.1002/elps.201000271

[35] Zhou L, Huang LQ, Beuerman RW, Grigg ME, et al (2004) Proteomic analysis of human tears: defensin expression after ocular surface surgery. J Proteome Res 3: 410–416. 15253421 10.1021/pr034065n

[36] Green-Church KB, Nichols KK, Kleinholz NM, Zhang L, Nichols JJ (2008) Investigation of the human tear film proteome using multiple proteomic approaches. Mol Vis 7:14: 456–470. PMC2268847 18334958

[37] Gachon AM, Verrelle P, Betail G, Dastugue B (1979) Immunological and electrophoretic studies of human tear proteins. Experimental Eye Research 29: 539–553. 118888 10.1016/0014-4835(79)90154-4

[38] Fullard RJ (1988) Identification of proteins in small tear volumes with and without size exclusion HPLC fractionation. Current Eye Research 7: 163–179. 2836132 10.3109/02713688808995746

[39] Kijlstra A, Kuizenga A (1994) Analysis and function of the human tear proteins. Advances in Experimental Medicine and Biology 350: 299–308. 8030492 10.1007/978-1-4615-2417-5_51

[40] Gachon A, Lacazette E (1998) Tear lipocalin and the eye's front line of defence. The British Journal of Ophthalmology 82: 453–455. 9640200 10.1136/bjo.82.4.453 PMC1722558

[41] Sitaramamma T, Shivaji S, Rao GN (1998) HPLC analysis of closed, open, and reflex eye tear proteins. Indian Journal of Ophthalmology 46: 239–245. 10218308

[42] Twining SS, Fukuchi T, Yue BY, Wilson PM, Boskovic G (1994) Corneal synthesis of alpha 1-proteinase inhibitor (alpha 1-antitrypsin). Investigative Ophthalmology & Visual Science 35: 458–462. 8112994

[43] Abrahamson M, Barrett AJ, Salvesen G, Grubb A (1986) Isolation of six cysteine proteinase inhibitors from human urine. Their physicochemical and enzyme kinetic properties and concentrations in biological fluids. The Journal of Biological Chemistry 261: 11282–11289. 3488317

[44] Barka T, Asbell PA, van der Noen H, Prasad A (1991) Cystatins in human tear fluid. Current Eye Research 10: 25–34. 2029847 10.3109/02713689109007608

[45] Isemura S, Saitoh E, Sanada K, Minakata K (1991) Identification of full-sized forms of salivary (S-type) cystatins (cystatin SN, cystatin SA, cystatin S, and two phosphorylated forms of cystatin S) in human whole saliva and determination of phosphorylation sites of cystatin S. Journal of Biochemistry 110: 648–654. 1778989 10.1093/oxfordjournals.jbchem.a123634

[46] Reitz C, Breipohl W, Augustin A, Bours J (1998) Analysis of tear proteins by one- and two-dimensional thin-layer iosoelectric focusing, sodium dodecyl sulfate electrophoresis and lectin blotting. Detection of a new component: cystatin C. Graefe's Archive for Clinical and Experimental Ophthalmology 236: 894–899. 10.1007/s004170050177 9865619

[47] Ananthi S, Santhosh RS, Nila MV, Prajna NV, Lalitha P, et al (2011) Comparative proteomics of human male and female tears by two-dimensional electrophoresis. Experimental Eye Research 92: 454–463. 21396361 10.1016/j.exer.2011.03.002

[48] ter Rahe BS, van Haeringen NJ (1998) Cystatins in tears of patients with different corneal conditions. Ophthalmologica 212: 34–36. 9438582 10.1159/000027256

[49] Ma P, Wang N, McKown RL, Raab RW, Laurie GW, et al (2006) Heparanase deglycanation of syndecan-1 is required for binding of the epithelial-restricted prosecretory mitogen lacritin. The Journal of Cell Biology 174: 1097–1106. 16982797 10.1083/jcb.200511134 PMC1666580

[50] Sanghi S, Kumar R, Lumsden A, Dickinson D, Klepeis V, et al (2001) cDNA and genomic cloning of lacritin, a novel secretion enhancing factor from the human lacrimal gland. Journal of Molecular Biology 310: 127–139. 11419941 10.1006/jmbi.2001.4748

[51] Samudre S, Lattanzio FA Jr, Lossen V, Hosseini A, Sheppard JD Jr, et al (2011) Lacritin, a novel human tear glycoprotein, promotes sustained basal tearing and is well tolerated. Invest Ophthalmol Vis Sci 5 52 9: 6265–6270. 10.1167/iovs.10-6220 PMC3176019 21087963

[52] Nichols JJ, Green-Church KB (2009) Mass spectrometry-based proteomic analyses in contact lens-related dry eye. Cornea 28: 1109–1117. 19770725 10.1097/ICO.0b013e3181a2ad81

[53] Breit SN, Wakefield D, Robinson JP, Luckhurst E, Clark P (1985) The role of alpha 1-antitrypsin deficiency in the pathogenesis of immune disorders. Clinical Immunology and Immunopathology 35: 363–380. 3886224 10.1016/0090-1229(85)90097-2

[54] Chevance LG, Causse JR, Bergès J (1976) Alpha 1-antitrypsin activity of perilymph. Occurrence during progression of otospongiosis. Arch Otolaryngol 102 6: 363–364. 1084149 10.1001/archotol.1976.00780110075008

[55] Kyaw-Myint TO, Howell AM, Murphy GM, Anderson CM (1975) Alpha-1-antitrypsin in duodenal fluid and gallbladder bile. Clin Chim Acta 22 59 1: 51–54. 10.1016/0009-8981(75)90217-x 1078995

[56] Talamo RC (1975) Basic and clinical aspects of the alpha1-antitrypsin. Pediatrics 56 1: 91–9. 1099521

[57] Sharp HL (1976) The current status of alpha-1-antityrpsin, a protease inhibitor, in gastrointestinal disease. Gastroenterology 70 4: 611–621. 767197

[58] Arora PK, Miller HC, Aronson LD (1978) Alpha1-Antitrypsin is an effector of immunological stasis. Nature 274: 589–590. 307694 10.1038/274589a0

[59] Berman MB, Barber JC, Talamo RC, Langley CE (1973) Corneal ulceration and the serum antiproteases. I. Alpha 1-antitrypsin. Investigative Ophthalmology 12: 759–770. 4361492

[60] Gupta AK, Sarin GS, Mathur MD, Ghosh B (1988) Alpha 1-antitrypsin and serum albumin in tear fluids in acute adenovirus conjunctivitis. The British Journal of Ophthalmology 72: 390–393. 2840112 10.1136/bjo.72.5.390 PMC1041461

[61] Prause JU (1983) Serum albumin, serum antiproteases and polymorphonuclear leucocyte neutral collagenolytic protease in the tear fluid of patients with corneal ulcers. Acta Ophthalmologica 61: 272–282. 6192677 10.1111/j.1755-3768.1983.tb01421.x

[62] Sen DK, Sarin GS (1986) The preocular tear film in health, disease and contact lens wear. Texas: Lubbock. Dry Eye Institute. pp 192–199.

[63] Sharp HL (1975) Modern trends in gastroenterology. 5th ed. London: Butterworths. 140 p.

[64] Langlois MR, Delanghe JR (1996) Biological and clinical significance of haptoglobin polymorphism in humans. Clin Chem 42: 1589–1600. 8855140

[65] Mii S, Nakamura K, Takeo K, Kurimoto S (1992) Analysis of human tear proteins by two-dimensional electrophoresis. Electrophoresis 13: 379–382. 1380449 10.1002/elps.1150130177

[66] Tseng CF, Lin CC, Huang HY, Liu HC, Mao SJT (2004) Antioxidant role of human haptoglobin. Proteomics 4: 2221–2228. 15274115 10.1002/pmic.200300787

[67] Tada T, Ohkubo I, Niwa M, Sasaki M, Tateyama H (1991) Immunohistochemical localization of Zn-alpha 2-glycoprotein in normal human tissues. The Journal of Histochemistry and Cytochemistry: Official Journal of the Histochemistry Society 39: 1221–1226. 1918940 10.1177/39.9.1918940

[68] Hirai K, Hussey HJ, Barber MD, Price SA, Tisdale MJ (1998) Biological evaluation of a lipid-mobilizing factor isolated from the urine of cancer patients. Cancer Res 1 58 11: 2359–2365. 9622075

[69] Russell ST, Zimmerman TP, Domin BA, Tisdale MJ (2004) Induction of lipolysis in vitro and loss of body fat in vivo by zinc-alpha2-glycoprotein. Biochimica Et Biophysica Acta 1636: 59–68. 14984739 10.1016/j.bbalip.2003.12.004

[70] Baker GRC, Morton M, Rajapaska RS, Bullock M, Gullu S, et al (2006) Altered tear composition in smokers and patients with graves ophthalmopathy. Archives of Ophthalmology 124: 1451–1456. 17030713 10.1001/archopht.124.10.1451

[71] Kawai S, Nakajima T, Hokari S, Komoda T, Kawai K (2002) Apolipoprotein A-I concentration in tears in diabetic retinopathy. Annals of Clinical Biochemistry 39: 56–61. 11853190 10.1258/0004563021901748

[72] Kolte RA, Kolte AP, Kohad RR (2010) Quantitative estimation and correlation of serum albumin levels in clinically healthy subjects and chronic periodontitis patients. Journal of Indian Society of Periodontology 14: 227–230. 21731247 10.4103/0972-124X.76923 PMC3118072

[73] Metz-Boutigue MH, Jollès J, Mazurier J, Schoentgen F, Legrand D, et al (1984) Human lactotransferrin: amino acid sequence and structural comparisons with other transferrins. European Journal of Biochemistry/FEBS 145: 659–676. 10.1111/j.1432-1033.1984.tb08607.x 6510420

[74] Kanyshkova TG, Buneva VN, Nevinsky GA (2001). Lactoferrin and Its biological functions. Biochemistry (Moscow) 66 p1-7-7. 10.1023/a:1002817226110 11240386

[75] Birgens HS (1985) Lactoferrin in plasma measured by an ELISA technique: evidence that plasma lactoferrin is an indicator of neutrophil turnover and bone marrow activity in acute leukaemia. Scandinavian Journal of Haematology 34: 326–331. 3858982 10.1111/j.1600-0609.1985.tb00757.x

[76] Brock JH (2002) The physiology of lactoferrin. Biochemistry and Cell Biology 80: 1–6. 10.1139/o01-212 11908632

[77] Bradford MM (1976) A rapid and sensitive method for the quantitation of microgram quantities of protein utilizing the principle of protein-dye binding. Analytical Biochemistry 72: 248–254. 942051 10.1016/0003-2697(76)90527-3

[78] Ramachandran B, Dikshit KL, Dharmalingam K (2012) Recombinant *E.coli* expressing *Vitreoscilla* haemoglobin prefers aerobic metabolism under microaerobic conditions: A proteome level study. J Biosci 37: 617–633. 22922188 10.1007/s12038-012-9245-z

[79] Neuhoff V, Stamm R, Eibl H (1985) Clear background and highly sensitive protein staining with Coomassie Blue dyes in polyacrylamide gels: A systematic analysis. Electrophoresis 6: 427–448.

[80] Huang DW, Sherman BT, Lempicki RA (2009) Systematic and integrative analysis of large gene lists using DAVID bioinformatics resources. Nature Protocols 4: 44–57. 19131956 10.1038/nprot.2008.211

