# Supplementary material for: Pathogen Induced Changes in the Protein Profile of Human Tears from Fusarium Keratitis Patients
Source: PLoS One. 2013 Jan 8;8(1):e53018. doi: 10.1371/journal.pone.0053018 (PMC3540078; doi:10.1371/journal.pone.0053018)
Supplement: Table S1 — Differentially Expressed Proteins in Tears of keratitis patients. (DOCX) [file pone.0053018.s001.docx]

Supplementary Table S1: Differentially Expressed Proteins in Tears of keratitis patients

| **Master No.** | **Identified Protein Name** | **Spot ID** | **Pattern Of Regulation** | **Fold variation Av. Ratio - Cy3+Cy5 gels** | **T-test** | **1-ANOVA** | **Fold variation Av. Ratio - Cy5 Gels** | **Fold variation Av. Ratio - Cy3 Gels** |
| --- | --- | --- | --- | --- | --- | --- | --- | --- |
| 1436 | Cystatin SA III precursor | 10 | Down regulation | -3.22 | 0.00029 | 0.0066 | -3.32 | -3.07 |
| 1207 | Haptoglobin α2 chain (M) | 19 | Up regulation | 4.93 | 0.0005 | 0.003 | 4.32 | 5.87 |
| 752 | Lacrimal Lipocalin precursor | 24 | Down regulation | -4.04 | 0.0038 | 0.037 | -4.05 | -4.03 |
| 1056 | Lacritin Precursor | 30 | Down regulation | -9.64 | 0.00036 | 0.00025 | -9.28 | -10.2 |
| 997 | Apolipoprotein | 32 | Up regulation | 5.19 | 0.0014 | 0.023 | 3.91 | 7.14 |
| 618 | Zinc-alpha-2-glycoprotein | 38 | Up regulation | 4.37 | 0.0032 | 0.043 | 2.87 | 7.59 |
| 628 | Haptoglobin precursor - β chain | 40 | Up regulation | 10.96 | 0.00024 | 0.0053 | 10.15 | 11.73 |
| 255 | Lactoferrin | 52 | Up regulation | 5.35 | 0.00088 | 0.0098 | 4.31 | 7.12 |
| 294 | Albumin | 58 | Up regulation | 4.56 | 0.0059 | 0.041 | 5.19 | 4.04 |
| 451 | Alpha-1-antitrypsin | 63 | Up regulation | 12.04 | 2.80E-05 | 0.00074 | 11.63 | 14.01 |
| 1057 |  |  |  | -12.18 | 6.40E-05 | 0.00069 | -10.51 | -12.99 |
| 986 |  |  |  | -8.52 | 0.00088 | 0.0085 | -9.59 | -7.07 |
| 1054 |  |  |  | -8.17 | 0.0024 | 0.0016 | -8.24 | -6.83 |
| 1097 |  |  |  | -7.43 | 0.0016 | 0.00099 | -6.57 | -8.75 |
| 1075 |  |  |  | -5.84 | 0.0027 | 0.0023 | -5.96 | -5.69 |
| 1080 |  |  |  | -5.7 | 0.02 | 0.0016 | -5.64 | -4.74 |
| 1142 |  |  |  | -5.3 | 0.029 | 0.045 | -7.55 | -4.77 |
| 1091 |  |  |  | -5.26 | 0.015 | 0.00028 | -4.73 | -6.11 |
| 1047 |  |  |  | -4.84 | 0.0069 | 0.00071 | -5.11 | -4.48 |
| 1189 |  |  |  | -4.14 | 0.054 | 0.037 | -3.34 | -4.87 |
| 654 |  |  |  | -4.06 | 0.0042 | 0.029 | -7.53 | -2.49 |
| 1349 |  |  |  | -3.93 | 0.03 | 0.038 | -7.25 | -3.01 |
| 948 |  |  |  | -3.88 | 0.001 | 0.0032 | -5.2 | -2.77 |
| 1326 |  |  |  | -3.78 | 0.058 | 0.024 | -3.78 | -3.78 |
| 1123 |  |  |  | -3.75 | 0.0048 | 0.022 | -4.09 | -3.75 |
| 1418 |  |  |  | -3.71 | 0.013 | 0.042 | -4.54 | -3.75 |
| 934 |  |  |  | -3.7 | 0.0028 | 0.017 | -3.99 | -3.27 |
| 1078 |  |  |  | -3.66 | 0.038 | 0.00074 | -3.78 | -3.47 |
| 1016 |  |  |  | -3.61 | 0.00051 | 0.011 | -4.61 | -2.81 |
| 713 |  |  |  | -3.6 | 0.0043 | 0.024 | -3.21 | -3.51 |
| 1013 |  |  |  | -3.55 | 0.028 | 0.019 | -5.92 | -2.61 |
| 648 |  |  |  | -3.47 | 0.00088 | 0.0083 | -5.09 | -2.42 |
| 764 |  |  |  | -3.32 | 0.012 | 0.0048 | -3.09 | -4.04 |
| 1098 |  |  |  | -3.29 | 0.013 | 0.0065 | -3.2 | -3.44 |
| 318 |  |  |  | -3.07 | 0.00015 | 0.0046 | -3.28 | -2.89 |
| 323 |  |  |  | -3.05 | 0.0019 | 0.023 | -3.41 | -2.74 |
| 1073 |  |  |  | -3.02 | 0.037 | 0.034 | -2.46 | -3.24 |
| 977 |  |  |  | -3.01 | 0.0074 | 0.037 | -3.85 | -2.18 |
| 1090 |  |  |  | -2.89 | 0.12 | 0.041 | -5.01 | -1.69 |
| 963 |  |  |  | -2.84 | 0.0038 | 0.017 | -2.35 | -3.16 |
| 866 |  |  |  | -2.76 | 0.064 | 0.032 | -1.4 | -4.99 |
| 309 |  |  |  | -2.7 | 0.00051 | 0.0046 | -2.75 | -2.66 |
| 315 |  |  |  | -2.69 | 0.00098 | 0.00099 | -2.5 | -2.9 |
| 457 |  |  |  | -2.66 | 0.029 | 0.025 | -2.16 | -3.3 |
| 1109 |  |  |  | -2.6 | 0.002 | 0.027 | -2.65 | 1.08 |
| 887 |  |  |  | -2.58 | 0.041 | 0.037 | -4 | -1.68 |
| 307 |  |  |  | -2.57 | 0.00023 | 0.0025 | -2.35 | -2.81 |
| 691 |  |  |  | -2.57 | 0.012 | 0.024 | -3.11 | -2.14 |
| 944 |  |  |  | -2.57 | 0.0016 | 0.0062 | -3.49 | -2.05 |
| 626 |  |  |  | -2.56 | 0.18 | 0.05 | -1.98 | -3.7 |
| 659 |  |  |  | -2.47 | 0.013 | 0.012 | -2.33 | -2.66 |
| 757 |  |  |  | -2.44 | 0.013 | 0.0096 | -2.39 | -2.28 |
| 1347 |  |  |  | -2.42 | 0.018 | 0.0051 | -2.24 | -2.64 |
| 259 |  |  |  | -2.4 | 0.093 | 0.02 | -2.08 | -2.76 |
| 317 |  |  |  | -2.35 | 0.015 | 0.0066 | -2.31 | -2.4 |
| 638 |  |  |  | -2.35 | 0.0065 | 0.011 | -2.97 | -1.93 |
| 774 |  |  |  | -2.26 | 0.0078 | 0.025 | -1.62 | -3.11 |
| 1367 |  |  |  | -2.26 | 0.014 | 0.025 | -2.14 | -2.35 |
| 760 |  |  |  | -2.25 | 0.072 | 0.037 | -1.77 | -2.9 |
| 639 |  |  |  | -2.24 | 0.002 | 0.0043 | -2.28 | -2.19 |
| 1462 |  |  |  | -2.22 | 0.0011 | 0.0042 | -1.9 | -2.61 |
| 613 |  |  |  | -2.2 | 0.0026 | 0.011 | -2.14 | -2.34 |
| 1070 |  |  |  | -2.16 | 0.14 | 0.042 | -1.96 | -2.17 |
| 1144 |  |  |  | -2.16 | 0.022 | 0.0023 | -2.88 | -1.7 |
| 238 |  |  |  | -2.14 | 0.016 | 0.025 | -2.9 | -1.62 |
| 1196 |  |  |  | -2.13 | 0.029 | 0.032 | -2.56 | -1.7 |
| 1463 |  |  |  | -2.13 | 0.0019 | 0.0043 | -2.15 | -2.11 |
| 800 |  |  |  | -2.11 | 0.0078 | 0.029 | -1.93 | -2.44 |
| 1352 |  |  |  | -2.1 | 0.08 | 0.027 | -2.59 | -1.7 |
| 167 |  |  |  | -2.08 | 0.042 | 0.032 | -2.6 | -1.63 |
| 177 |  |  |  | -2.06 | 0.014 | 0.0072 | -2.17 | -1.84 |
| 310 |  |  |  | -2.05 | 0.00015 | 0.00099 | -2.06 | -2.04 |
| 1508 |  |  |  | -2.05 | 0.00098 | 0.0065 | -1.78 | -2.21 |
| 230 |  |  |  | -2.04 | 0.034 | 0.027 | -2.66 | -1.64 |
| 1181 |  |  |  | -2.02 | 0.092 | 0.032 | -3.24 | -1.19 |
| 1466 |  |  |  | -1.99 | 0.044 | 0.014 | -1.83 | -2.15 |
| 1487 |  |  |  | -1.98 | 0.017 | 0.0078 | -1.77 | -2.25 |
| 1495 |  |  |  | -1.98 | 0.054 | 0.032 | -2.15 | -1.83 |
| 771 |  |  |  | -1.93 | 0.013 | 0.01 | -1.99 | -1.87 |
| 199 |  |  |  | -1.92 | 0.0074 | 0.041 | -2.15 | -1.72 |
| 1396 |  |  |  | -1.88 | 0.055 | 0.011 | -1.44 | -2.58 |
| 1441 |  |  |  | -1.88 | 0.018 | 0.025 | -1.79 | -1.76 |
| 320 |  |  |  | -1.84 | 0.11 | 0.025 | -1.77 | -1.74 |
| 325 |  |  |  | -1.84 | 0.17 | 0.048 | -3.05 | -1.37 |
| 312 |  |  |  | -1.79 | 0.0013 | 0.02 | -1.9 | -1.69 |
| 1464 |  |  |  | -1.77 | 0.11 | 0.045 | -1.17 | -2.89 |
| 1180 |  |  |  | -1.73 | 0.2 | 0.012 | -2.28 | -1.33 |
| 1361 |  |  |  | -1.7 | 0.12 | 0.024 | -1.9 | -1.36 |
| 311 |  |  |  | -1.67 | 0.01 | 0.0092 | -1.55 | -1.88 |
| 754 |  |  |  | -1.62 | 0.17 | 0.037 | -1.86 | -1.34 |
| 895 |  |  |  | -1.6 | 0.098 | 0.02 | -1.46 | -1.78 |
| 1471 |  |  |  | -1.6 | 0.16 | 0.011 | -1.46 | -1.78 |
| 285 |  |  |  | -1.59 | 0.092 | 0.0025 | -1.51 | -1.66 |
| 194 |  |  |  | -1.55 | 0.12 | 0.049 | -1.16 | -2.04 |
| 1229 |  |  |  | -1.55 | 0.2 | 0.0042 | -1.68 | -1.42 |
| 1427 |  |  |  | -1.54 | 0.069 | 0.048 | -1.88 | -1.32 |
| 453 |  |  |  | -1.53 | 0.1 | 0.027 | -2.25 | -1.13 |
| 622 |  |  |  | 1.62 | 0.07 | 0.042 | 2.19 | 1.4 |
| 189 |  |  |  | 1.71 | 0.026 | 0.04 | 1.72 | 1.73 |
| 979 |  |  |  | 1.76 | 0.022 | 0.032 | 2.14 | 1.44 |
| 297 |  |  |  | 1.8 | 0.014 | 0.0097 | 1.65 | 1.95 |
| 193 |  |  |  | 1.81 | 0.048 | 0.0025 | 1.84 | 1.77 |
| 569 |  |  |  | 1.81 | 0.039 | 0.024 | 1.85 | 1.7 |
| 96 |  |  |  | 1.94 | 0.0094 | 0.043 | 1.65 | 2.33 |
| 355 |  |  |  | 1.95 | 0.66 | 0.042 | 1.84 | 2.59 |
| 492 |  |  |  | 2.06 | 0.097 | 0.027 | 2.28 | 1.51 |
| 604 |  |  |  | 2.07 | 0.048 | 0.015 | 2.01 | 1.98 |
| 290 |  |  |  | 2.09 | 0.41 | 0.01 | 1.71 | 3.73 |
| 197 |  |  |  | 2.1 | 0.0048 | 0.0081 | 2 | 2.11 |
| 464 |  |  |  | 2.14 | 0.00088 | 0.006 | 1.84 | 2.54 |
| 528 |  |  |  | 2.17 | 0.24 | 0.011 | 2.18 | 2.16 |
| 466 |  |  |  | 2.22 | 0.0026 | 0.017 | 2.62 | 1.87 |
| 745 |  |  |  | 2.28 | 0.29 | 0.01 | 2.2 | 2.3 |
| 551 |  |  |  | 2.32 | 0.0018 | 0.028 | 1.91 | 2.74 |
| 470 |  |  |  | 2.34 | 0.019 | 0.02 | 2.18 | 2.53 |
| 100 |  |  |  | 2.35 | 0.00043 | 0.0092 | 2.5 | 2.16 |
| 424 |  |  |  | 2.38 | 0.072 | 0.037 | 2.15 | 2.67 |
| 1453 |  |  |  | 2.4 | 0.66 | 0.0036 | 3.46 | 1.57 |
| 1469 |  |  |  | 2.44 | 0.0019 | 0.017 | 2.55 | 2.29 |
| 899 |  |  |  | 2.47 | 0.003 | 0.0099 | 2.07 | 3.07 |
| 976 |  |  |  | 2.49 | 0.00082 | 0.0084 | 2.41 | 2.59 |
| 269 |  |  |  | 2.5 | 0.005 | 0.033 | 2.33 | 2.9 |
| 826 |  |  |  | 2.52 | 0.0082 | 0.032 | 1.92 | 3.63 |
| 196 |  |  |  | 2.7 | 0.022 | 0.027 | 2.29 | 3.47 |
| 956 |  |  |  | 2.7 | 0.00013 | 0.0012 | 3.32 | 2.21 |
| 915 |  |  |  | 2.71 | 0.0016 | 0.002 | 2.78 | 2.63 |
| 305 |  |  |  | 2.72 | 0.032 | 0.037 | 3.74 | 3.22 |
| 890 |  |  |  | 2.72 | 0.0095 | 0.023 | 2.35 | 3.02 |
| 926 |  |  |  | 2.73 | 0.0055 | 0.017 | 2.56 | 2.94 |
| 932 |  |  |  | 2.73 | 0.00043 | 0.0066 | 2.75 | 2.71 |
| 1003 |  |  |  | 2.76 | 0.00061 | 0.0098 | 2.63 | 2.91 |
| 694 |  |  |  | 2.83 | 0.0029 | 0.0016 | 2.8 | 2.85 |
| 924 |  |  |  | 2.84 | 0.002 | 0.0092 | 3.05 | 2.61 |
| 925 |  |  |  | 2.93 | 0.00079 | 0.0047 | 2.99 | 2.78 |
| 914 |  |  |  | 2.95 | 0.00011 | 0.00021 | 3.15 | 2.75 |
| 95 |  |  |  | 2.98 | 0.0033 | 0.032 | 2.03 | 4.67 |
| 988 |  |  |  | 2.98 | 0.0055 | 0.011 | 2.99 | 2.82 |
| 623 |  |  |  | 3.03 | 0.0064 | 0.00023 | 2.57 | 3.68 |
| 973 |  |  |  | 3.07 | 0.00043 | 0.0042 | 2.8 | 3.39 |
| 900 |  |  |  | 3.13 | 0.0069 | 0.049 | 4.45 | 2.2 |
| 503 |  |  |  | 3.15 | 0.16 | 0.027 | 2.53 | 3.74 |
| 931 |  |  |  | 3.15 | 0.00058 | 0.002 | 3.67 | 2.48 |
| 632 |  |  |  | 3.16 | 8.60E-05 | 0.0012 | 2.99 | 3.38 |
| 949 |  |  |  | 3.16 | 0.00081 | 0.0022 | 3.59 | 2.89 |
| 103 |  |  |  | 3.22 | 0.04 | 0.05 | 3.6 | 3.59 |
| 960 |  |  |  | 3.23 | 0.00018 | 0.0034 | 3.71 | 2.78 |
| 291 |  |  |  | 3.24 | 0.24 | 0.046 | 1.98 | 5.73 |
| 892 |  |  |  | 3.32 | 0.00052 | 0.0029 | 3.33 | 3.3 |
| 768 |  |  |  | 3.35 | 0.01 | 0.025 | 3.09 | 3.12 |
| 716 |  |  |  | 3.45 | 0.0016 | 0.02 | 3.12 | 3.34 |
| 642 |  |  |  | 3.46 | 0.00084 | 0.014 | 3.27 | 3.65 |
| 560 |  |  |  | 3.47 | 0.0033 | 0.013 | 3.87 | 3.2 |
| 652 |  |  |  | 3.49 | 0.00095 | 0.0092 | 3.3 | 3.7 |
| 248 |  |  |  | 3.52 | 0.007 | 0.015 | 2.97 | 4.37 |
| 53 |  |  |  | 3.54 | 0.0015 | 0.011 | 3.44 | 3.63 |
| 586 |  |  |  | 3.58 | 0.012 | 0.038 | 4.69 | 2.78 |
| 1288 |  |  |  | 3.58 | 0.0051 | 0.025 | 4.4 | 2.97 |
| 404 |  |  |  | 3.65 | 0.0043 | 0.029 | 4.22 | 3.14 |
| 1004 |  |  |  | 3.69 | 0.0057 | 0.048 | 3.3 | 4.08 |
| 253 |  |  |  | 3.75 | 0.00088 | 0.0092 | 3.48 | 4.19 |
| 399 |  |  |  | 3.75 | 0.046 | 0.034 | 3.29 | 4.14 |
| 1012 |  |  |  | 3.79 | 0.0026 | 0.035 | 3.02 | 4.95 |
| 460 |  |  |  | 3.8 | 0.01 | 0.041 | 3.7 | 3.67 |
| 666 |  |  |  | 3.8 | 0.00013 | 0.0025 | 4.02 | 3.48 |
| 1373 |  |  |  | 3.83 | 0.48 | 0.0029 | 4.48 | 3.33 |
| 805 |  |  |  | 3.86 | 0.00058 | 0.011 | 3.03 | 5.41 |
| 210 |  |  |  | 3.88 | 0.0082 | 0.027 | 2.95 | 5.6 |
| 651 |  |  |  | 3.9 | 0.00088 | 0.012 | 4.47 | 3.56 |
| 704 |  |  |  | 3.91 | 0.00081 | 0.0097 | 3.98 | 3.8 |
| 913 |  |  |  | 3.98 | 4.60E-05 | 0.00071 | 3.8 | 4.19 |
| 907 |  |  |  | 4.01 | 0.0038 | 0.023 | 3.85 | 3.71 |
| 993 |  |  |  | 4.06 | 0.00015 | 0.0036 | 4.64 | 3.53 |
| 595 |  |  |  | 4.08 | 0.0033 | 0.0055 | 3.74 | 4.8 |
| 1039 |  |  |  | 4.16 | 0.001 | 0.014 | 3.97 | 4.99 |
| 991 |  |  |  | 4.25 | 0.0061 | 0.032 | 5.61 | 3.06 |
| 735 |  |  |  | 4.27 | 0.0025 | 0.01 | 2.99 | 6.11 |
| 360 |  |  |  | 4.35 | 0.043 | 0.05 | 4.13 | 3.11 |
| 247 |  |  |  | 4.59 | 0.0025 | 0.019 | 3.66 | 6.2 |
| 995 |  |  |  | 4.62 | 0.00025 | 0.005 | 4.44 | 4.87 |
| 388 |  |  |  | 4.66 | 0.003 | 0.032 | 5.6 | 4.19 |
| 675 |  |  |  | 4.68 | 0.00016 | 0.0049 | 5.11 | 4.23 |
| 467 |  |  |  | 4.79 | 1.60E-05 | 0.00071 | 5.2 | 4.47 |
| 409 |  |  |  | 4.82 | 0.00015 | 0.0015 | 4.12 | 5.75 |
| 413 |  |  |  | 4.83 | 0.0012 | 0.015 | 4.57 | 5.13 |
| 514 |  |  |  | 4.85 | 0.0015 | 0.024 | 5.41 | 4.3 |
| 403 |  |  |  | 4.92 | 0.018 | 0.02 | 5.05 | 4.84 |
| 496 |  |  |  | 4.98 | 0.0011 | 0.017 | 4.13 | 5.92 |
| 398 |  |  |  | 4.98 | 0.0019 | 0.0095 | 3.31 | 7.17 |
| 635 |  |  |  | 5 | 0.00047 | 0.002 | 5.75 | 4.57 |
| 272 |  |  |  | 5.03 | 0.0016 | 0.025 | 7.65 | 3.24 |
| 132 |  |  |  | 5.06 | 0.00043 | 0.009 | 4.9 | 5.24 |
| 610 |  |  |  | 5.12 | 2.30E-05 | 0.00099 | 6.1 | 4.33 |
| 99 |  |  |  | 5.15 | 0.00088 | 0.0041 | 3.71 | 7.21 |
| 256 |  |  |  | 5.19 | 0.0024 | 0.017 | 4.33 | 6.37 |
| 449 |  |  |  | 5.2 | 0.0024 | 0.011 | 8.34 | 4.49 |
| 352 |  |  |  | 5.22 | 0.0051 | 0.032 | 7.05 | 3.74 |
| 516 |  |  |  | 5.28 | 5.30E-05 | 0.0022 | 4.94 | 5.54 |
| 432 |  |  |  | 5.37 | 0.0071 | 0.024 | 7.12 | 4.36 |
| 689 |  |  |  | 5.39 | 0.00029 | 0.0063 | 4.76 | 6.07 |
| 1212 |  |  |  | 5.4 | 0.0024 | 0.014 | 3.79 | 9.14 |
| 608 |  |  |  | 5.52 | 0.0038 | 0.05 | 9.6 | 4.09 |
| 510 |  |  |  | 5.55 | 0.00092 | 0.015 | 5.8 | 5.29 |
| 990 |  |  |  | 5.57 | 0.0095 | 0.043 | 7.02 | 4.67 |
| 1030 |  |  |  | 5.61 | 0.00012 | 0.0036 | 5.22 | 6.5 |
| 703 |  |  |  | 5.71 | 0.00082 | 0.0092 | 4.7 | 6.73 |
| 105 |  |  |  | 5.77 | 0.0056 | 0.043 | 5.1 | 9.75 |
| 705 |  |  |  | 5.83 | 0.00012 | 0.0015 | 5.01 | 6.43 |
| 133 |  |  |  | 5.88 | 0.00093 | 0.0065 | 5.31 | 7.05 |
| 725 |  |  |  | 5.9 | 0.0069 | 0.00054 | 7.14 | 4.84 |
| 557 |  |  |  | 5.92 | 0.00046 | 0.0065 | 5.31 | 6.8 |
| 366 |  |  |  | 6.18 | 0.0043 | 0.023 | 10.94 | 5.36 |
| 462 |  |  |  | 6.2 | 0.00011 | 0.0025 | 7.41 | 5.44 |
| 1008 |  |  |  | 6.31 | 0.00029 | 0.0066 | 7.11 | 5.54 |
| 275 |  |  |  | 6.38 | 0.0051 | 0.05 | 11.08 | 2.84 |
| 419 |  |  |  | 6.47 | 0.016 | 0.00074 | 6.3 | 6.62 |
| 481 |  |  |  | 6.49 | 0.00041 | 0.0084 | 4.88 | 8.19 |
| 102 |  |  |  | 6.58 | 0.0044 | 0.0097 | 8.27 | 8.52 |
| 494 |  |  |  | 6.75 | 0.00011 | 0.0036 | 5.97 | 7.88 |
| 1001 |  |  |  | 6.79 | 3.60E-05 | 0.00092 | 7.01 | 6.55 |
| 513 |  |  |  | 6.99 | 0.0047 | 0.035 | 11.62 | 4.84 |
| 70 |  |  |  | 7.04 | 0.003 | 0.022 | 4.2 | 12.15 |
| 1049 |  |  |  | 7.14 | 0.00015 | 0.0047 | 8.23 | 6.04 |
| 515 |  |  |  | 7.17 | 0.00022 | 0.0065 | 7.52 | 6.75 |
| 942 |  |  |  | 7.17 | 0.00042 | 0.0065 | 8.05 | 5.32 |
| 408 |  |  |  | 7.22 | 0.0075 | 0.0076 | 6.45 | 7.79 |
| 391 |  |  |  | 7.24 | 0.00029 | 0.0043 | 6.77 | 7.63 |
| 1005 |  |  |  | 7.44 | 2.20E-05 | 0.00099 | 7.92 | 7.09 |
| 646 |  |  |  | 7.51 | 0.00052 | 0.0072 | 6.65 | 8.59 |
| 501 |  |  |  | 7.59 | 3.30E-05 | 0.0012 | 8.01 | 7.12 |
| 615 |  |  |  | 7.65 | 0.0014 | 0.0033 | 11.02 | 4.46 |
| 384 |  |  |  | 7.88 | 0.0012 | 0.011 | 6.89 | 11.67 |
| 101 |  |  |  | 7.9 | 0.0048 | 0.021 | 7.45 | 8.51 |
| 570 |  |  |  | 8.15 | 0.00056 | 0.01 | 6.37 | 11.77 |
| 422 |  |  |  | 8.16 | 0.0014 | 0.014 | 14.84 | 6.19 |
| 600 |  |  |  | 8.36 | 0.0033 | 0.041 | 9.71 | 6.93 |
| 411 |  |  |  | 8.4 | 0.0059 | 0.0066 | 6.11 | 11.21 |
| 455 |  |  |  | 8.54 | 0.00013 | 0.0016 | 7.67 | 10.34 |
| 574 |  |  |  | 8.54 | 0.00081 | 0.013 | 6.62 | 14.87 |
| 1022 |  |  |  | 8.56 | 0.0015 | 0.018 | 10.87 | 6.78 |
| 576 |  |  |  | 8.75 | 0.00066 | 0.015 | 5.3 | 17.19 |
| 553 |  |  |  | 9 | 0.00093 | 0.02 | 8.06 | 9.77 |
| 518 |  |  |  | 9.02 | 3.60E-05 | 0.00094 | 9 | 9.03 |
| 523 |  |  |  | 9.28 | 0.00044 | 0.0098 | 16.56 | 6.45 |
| 362 |  |  |  | 9.54 | 0.00064 | 0.0088 | 7.86 | 10.91 |
| 69 |  |  |  | 9.55 | 0.00088 | 0.01 | 10.6 | 9.68 |
| 415 |  |  |  | 9.61 | 0.0038 | 0.0012 | 8.89 | 10.18 |
| 97 |  |  |  | 9.81 | 0.0019 | 0.023 | 17.9 | 7.54 |
| 531 |  |  |  | 9.84 | 0.00022 | 0.0065 | 7.2 | 15.95 |
| 64 |  |  |  | 9.98 | 0.0094 | 0.032 | 6.34 | 19.98 |
| 558 |  |  |  | 10.01 | 0.00011 | 0.0012 | 10.26 | 9.55 |
| 582 |  |  |  | 10.02 | 0.00013 | 0.0042 | 9.19 | 11.14 |
| 137 |  |  |  | 10.74 | 0.00012 | 0.0016 | 12.63 | 9.27 |
| 585 |  |  |  | 10.74 | 8.20E-05 | 0.0029 | 8.44 | 13.92 |
| 429 |  |  |  | 10.97 | 2.20E-05 | 0.00085 | 9.65 | 12.23 |
| 450 |  |  |  | 11.52 | 9.00E-06 | 0.00035 | 9.72 | 13.45 |
| 552 |  |  |  | 11.52 | 0.0012 | 0.01 | 12.23 | 11.01 |
| 566 |  |  |  | 11.79 | 1.60E-05 | 0.00071 | 12.86 | 11.07 |
| 521 |  |  |  | 12.38 | 2.20E-05 | 0.00099 | 9.46 | 17.27 |
| 538 |  |  |  | 12.94 | 0.00021 | 0.0047 | 9.15 | 22.9 |
| 387 |  |  |  | 13.12 | 0.00066 | 0.0066 | 14.05 | 13.24 |
| 1018 |  |  |  | 13.34 | 1.60E-05 | 0.00017 | 16.21 | 10.69 |
| 444 |  |  |  | 13.35 | 0.00081 | 0.0092 | 18.49 | 13.77 |
| 67 |  |  |  | 13.46 | 0.00088 | 0.011 | 13.86 | 13.56 |
| 529 |  |  |  | 13.58 | 4.70E-05 | 0.0019 | 13.71 | 13.48 |
| 1002 |  |  |  | 13.78 | 0.00012 | 0.0042 | 16.35 | 11.96 |
| 454 |  |  |  | 13.96 | 5.60E-06 | 0.00023 | 14.98 | 13.16 |
| 276 |  |  |  | 14.16 | 0.00066 | 0.0065 | 10.38 | 22.18 |
| 520 |  |  |  | 14.49 | 2.20E-05 | 0.0011 | 15.44 | 13.77 |
| 74 |  |  |  | 15.15 | 0.00041 | 0.0025 | 11.38 | 20.48 |
| 1020 |  |  |  | 15.32 | 3.60E-06 | 0.00023 | 14.82 | 15.96 |
| 1006 |  |  |  | 15.47 | 3.60E-06 | 0.00017 | 17.45 | 13.47 |
| 1025 |  |  |  | 15.77 | 1.70E-05 | 0.00071 | 13.79 | 18.72 |
| 66 |  |  |  | 16.37 | 0.00014 | 0.0025 | 10.62 | 25.32 |
| 534 |  |  |  | 16.54 | 2.00E-05 | 0.00085 | 15.25 | 18.18 |
| 1009 |  |  |  | 16.93 | 3.60E-06 | 0.00017 | 20.84 | 13.7 |
| 361 |  |  |  | 17.43 | 0.00013 | 0.0016 | 12.85 | 22.7 |
| 65 |  |  |  | 17.62 | 0.00028 | 0.0041 | 10.63 | 29.54 |
| 1028 |  |  |  | 17.9 | 0.00025 | 0.0065 | -1.2 | 12.29 |
| 1010 |  |  |  | 19.62 | 0.015 | 0.043 | 38.75 | 9.93 |
| 425 |  |  |  | 22.27 | 0.00043 | 0.0047 | 17.22 | 29.16 |
| 359 |  |  |  | 22.68 | 2.20E-05 | 0.00056 | 19.55 | 25.62 |
