# Supplementary material for: Pathogen Induced Changes in the Protein Profile of Human Tears from Fusarium Keratitis Patients
Source: PLoS One. 2013 Jan 8;8(1):e53018. doi: 10.1371/journal.pone.0053018 (PMC3540078; doi:10.1371/journal.pone.0053018)
Supplement: Table S2 — Identified Tear proteins and their functions. (DOCX) [file pone.0053018.s002.docx]

Supplementary Table S2: Identified Tear proteins and their functions

| **Spot ID** | **Identified Tear proteins** | **Database/Accession no.** | **Mowse score**^a^ | **X & Y coordinate in DIGE gel** | **M*r*/ p*I* 2-D^b^** | **M*r*/ p*I* data base^c^** | **Peptides Matched^d^** | **Sequence Coverage (%)^e^** | **Function** |
| --- | --- | --- | --- | --- | --- | --- | --- | --- | --- |
|  | Mammaglobin-B precursor | Swissprot/[SG2A1_HUMAN](http://www.matrixscience.com/cgi/protein_view.pl?file=../data/20080514/FtgpIbueL.dat&hit=SG2A1_HUMAN&px=1&ave_thresh=30&_sigthreshold=0.05&_server_mudpit_switch=0.001) | 255 (29) | 105,940 | < 14/5.1 | 10.8/5.48 | **K.LLEDMVEK.T K.TINSDISIPEYK.E K.ELLQEFIDSDAAAEAMGK.F** | 40 | To bind androgens and other steroids |
|  | Cystatin SA III potential precursor of acquired enemal | NCBI/ [gi\|235948](http://www.matrixscience.com/cgi/protein_view.pl?file=../data/20060606/FAGtfbSw.dat&hit=3) | 105 (27) | 92,920 | 14/4.5 | 14.1/4.74 | **R.IIPGGIYDADLNDEWVQR.A**  **R.ALHFAISEYNK.A K.ATEDEYYR.R R.RPLQVLR.A R.EQTFGGVNYFFDVEVGR.T K.SQPNLDTCAFHEQPELQK.K K.QLCSFEIYEVPWEDR.M** | 77 | Cysteine protease inhibitor |
|  | Cystatin S precursor | NCBI/ [gi\|4503109](http://www.matrixscience.com/cgi/protein_view.pl?file=../data/20080514/FtgpIbuaE.dat&hit=gi%7c4503109&px=1&ave_thresh=38&_sigthreshold=0.05&_server_mudpit_switch=0.001) | 60 (35) | 166,875 | 16/4.6 | 16.2/ 4.95 | **R.ALHFAISEYNK.A** | 7 | Cysteine protease inhibitor |
|  | Lysozyme-C precursor | MSDB/[LZHU](http://www.matrixscience.com/cgi/protein_view.pl?file=../data/20080514/FtgpIbsee.dat&hit=LZHU&px=1&ave_thresh=35&_sigthreshold=0.05&_server_mudpit_switch=0.001) | 81 (34) | 190,869 | 16/4.7 | 16.5/9.3 | **R.STDYGIFQINSR.Y** | 8 | Hydrolyzes polysaccharides found in many bacterial cell walls |
|  | Cystatin S precursor | MSDB / [UDHUP1](http://www.matrixscience.com/cgi/protein_view.pl?file=../data/20080514/FtgpIeaOE.dat&hit=UDHUP1&px=1&ave_thresh=36&_sigthreshold=0.05&_server_mudpit_switch=0.001) | 495 (36) | 242,870 | 16/4.8 | 16.2/ 4.95 | **R.IIPGGIYDADLNDEWVQR.A R.ALHFAISEYNK.A K.ATEDEYYRRPLQVLR.A K.SQPNLDTCAFHEQPELQK.K K.KQLCSFEIYEVPWEDR.M K.QLCSFEIYEVPWEDR.M** | 55 | Cysteine protease inhibitor |
|  | Chain B,crystal structure of component formed between Mhc like Zn alpha 2 glycoprotein and PIP | NCBI/[gi\|210061077](http://www.matrixscience.com/cgi/protein_view.pl?file=../data/20110826/FtoAibTnL.dat&hit=gi%7c210061077&db_idx=1&px=1&ave_thresh=38&_ignoreionsscorebelow=0&report=20&_sigthreshold=0.05&_msresflags=1025&_msresflags2=2&percolate=-1&percolate_rt=0) | 303 (38) | 375,870 | 14/5 | 13.5/5.4 | **K.IIIKNFDIPK.S K.NFDIPK.S K.YTACLCDDNPK.T K.TFYWDFYTNR.T R.TVQIAAVVDVIR.E R.FYTIEILKVE.-** | 44 | unknown |
|  | Lipocalin 1 precursor | NCBI /[gi\|4504963](http://www.matrixscience.com/cgi/protein_view.pl?file=../data/20080514/FtgpIbswh.dat&hit=gi%7c4504963&px=1&ave_thresh=38&_sigthreshold=0.05&_server_mudpit_switch=0.001) | 112 (28) | 474,909 | 14/5.2 | 19.2/5.39 | **K.NNLEALEDFEK.A R.GLSTESILIPR.Q** | 12 | Lipid scavenging and transport to outer tear layer |
|  | Chain A, Role Of Amino Acid Residues At Turns In The Conformational Stability And Folding Of Human Lysozyme | NCBI / [gi\|6730358](http://www.matrixscience.com/cgi/protein_view.pl?file=../data/20091117/FtmcmxYeS.dat&hit=gi%7c6730358&px=1&ave_thresh=36&_sigthreshold=0.05&_server_mudpit_switch=0.001) | 107 (36) | 453,830 | 14/5.5 | 14.9 / 9.14 | **K.RLGMDGYR.G R.LGMDGYR.G R.STDYGIFQINSR.Y K.RVVDPQGIR.A R.AWVAWR.N** | 27 | Hydrolyzes polysaccharides found in many bacterial cell walls |
|  | Transthyretin OS=Homo sapiens GN=TTR PE=1 SV=1 | Swissprot / [TTHY_HUMAN](http://www.matrixscience.com/cgi/protein_view.pl?file=../data/20091117/FtmcmzHmL.dat&hit=TTHY_HUMAN&px=1&ave_thresh=27&_sigthreshold=0.05&_server_mudpit_switch=0.001) | 361 (27) | 478,811 | 14/5.7 | 15.9/5.52 | **R.GSPAINVAVHVFR.K R.GSPAINVAVHVFRK.A R.GSPAINVAVHVFRK.A R.KAADDTWEPFASGK.T K.AADDTWEPFASGK.T** | 18 | Binds retinol to retinol-binding protein |
|  | **cystatin SA-III=potential precursor of acquired enamel pellicle [human, Peptide, 121 aa]** | **NCBI/**[**gi\|235948**](http://www.matrixscience.com/cgi/protein_view.pl?file=../data/20100625/FteofGESL.dat&hit=gi%7c235948&db_idx=1&px=1&ave_thresh=38&_ignoreionsscorebelow=0&report=20&_sigthreshold=0.05&_msresflags=1025&_msresflags2=2&percolate=-1&percolate_rt=0) | **182(38)** | **484,835** | **16/5.4** | **14.1/4.7** | **R.IIPGGIYDADLNDEWVQR.A R.RPLQVLR.A K.SQPNLDTCAFHEQPELQK.KK.SQPNLDTCAFHEQPELQK.K** | **35** | **Cysteine protease inhibitor** |
|  | Transthyretin OS=Homo sapiens GN=TTR PE=1 SV=1 | Swissprot/[TTHY_HUMAN](http://www.matrixscience.com/cgi/protein_view.pl?file=../data/20110826/FtoAibTam.dat&hit=TTHY_HUMAN&db_idx=1&px=1&ave_thresh=29&_ignoreionsscorebelow=0&report=20&_sigthreshold=0.05&_msresflags=1089&_msresflags2=2&percolate=-1&percolate_rt=0) | 60(27) | 536,835 | 14/5.8 | 15.8/5.52 | **R.GSPAINVAVHVFR.K** | 8 | Binds retinol to retinol-binding protein |
|  | Chain A, Cyrstal structure of Human tear lipocalinVON EBNERS GLAND Protein | NCBI/ [gi\|56554584](http://www.matrixscience.com/cgi/protein_view.pl?file=../data/20110222/FttolzueT.dat&hit=gi%7c56554584&db_idx=1&px=1&ave_thresh=37&_ignoreionsscorebelow=0&report=20&_sigthreshold=0.05&_msresflags=1025&_msresflags2=2&percolate=-1&percolate_rt=0) | 125 (37) | 566,892 | 14/5.7 | 17.9/5.27 | **K.VTMLISGR.C K.YTADGGKHVAYIIR.S K.HVAYIIR.S R.GLSTESILIPR.Q** | 20 | Lipid scavenging and transport to outer tear layer |
|  | **alpha-1-B-glycoprotein - human** | NCBI/[gi\|69990](http://www.matrixscience.com/cgi/protein_view.pl?file=../data/20100625/FteofasOT.dat&hit=gi%7c69990&db_idx=1&px=1&ave_thresh=39&_ignoreionsscorebelow=0&report=20&_sigthreshold=0.05&_msresflags=1025&_msresflags2=2&percolate=-1&percolate_rt=0) | 408(38) | 717,882. | 15/6.3 | 51.9/5.6 | **R.LETPDFQLFK.N K.LLELTGPK.S**  **K.LLELTGPK.S**  **K.LLELTGPK.S**  **K.LLELTGPK.S**  **R.GVTFLLR.R K.VTLTCVAPLSGVDFQLR.R**  **K.VTLTCVAPLSGVDFQLRR.G K.ELLVPR.S**  **R.CLAPLEGAR.F**  **R.LELHVDGPPPRPQLR.A**  **R.CEGPIPDVTFELLR.E**  **R.TPGAAANLELIFVGPQHAGNYR.C** | **22** | Unknown |
|  | RecName: Full=Cystatin-SN; AltName: Full=Cystatin-1; AltName: Full=Salivary cystatin-SA-1; AltName: Full=Cystain-SA-I; Flags: Precursor | NCBI/[gi\|337752](http://www.matrixscience.com/cgi/protein_view.pl?file=../data/20110826/FtoAibewS.dat&hit=gi%7c337752&db_idx=1&px=1&ave_thresh=35&_ignoreionsscorebelow=0&report=20&_sigthreshold=0.05&_msresflags=1025&_msresflags2=2&percolate=-1&percolate_rt=0) | 42(37) | 698,919 | 13/6.5 | 16.3/6.8 | **K.SQPNLDTCAFHEQPELQK.K** | 12 | Cysteine protease inhibitor |
|  | Beta-2-Microglobulin precursor | Swissprot//[B2MG_HUMAN](http://www.matrixscience.com/cgi/protein_view.pl?file=../data/20110223/FttoIfHOt.dat&hit=B2MG_HUMAN&db_idx=1&px=1&ave_thresh=46&_ignoreionsscorebelow=0&report=20&_sigthreshold=0.05&_msresflags=1025&_msresflags2=2&percolate=-1&percolate_rt=0) | 59 (28) | 728,917 | < 14/6.5 | 13.8/6.06 | **R.IEKVEHSDLSFSK.D R.VNHVTLSQPK.I** | 19 | Presentation of peptide antigens to the immune system. |
|  | Cystatin SN precursor | **NCBI/** [gi\|19882251](http://www.matrixscience.com/cgi/protein_view.pl?file=../data/20060707/FAEuSGEE.dat&hit=1) | 66 (55) | 827,902 | 15.7/8.48 | 16.3/ 6.7 | **R.IIPGGIYNADLNDEWVQR.A R.QQTVGGVNYFFDVEVGR.T K.SQPNLDTCAFHEQPELQK.K K.KQLCSFEIYEVPWENR.R K.QLCSFEIYEVPWENR.R K.QLCSFEIYEVPWENRR.S** | 49 | Cysteine protease inhibitor |
|  | Mutant lysozyme | NCBI/[gi\|1827553](http://www.matrixscience.com/cgi/protein_view.pl?file=../data/20110826/FtoAibHSh.dat&hit=gi%7c1827553&db_idx=1&px=1&ave_thresh=35&_ignoreionsscorebelow=0&report=20&_sigthreshold=0.05&_msresflags=1025&_msresflags2=2&percolate=-1&percolate_rt=0) | 121(36) | 765,770 | 14.3/6.7 | 14.6/9.3 | **R.LGMDGYR.G R.STDYGIFQINSR.Y R.AWVAWR.N** | 19 | Hydrolyzes polysaccharides found in many bacterial cell walls |
|  | Haptoglobin OS=Homo sapiens GN=HP PE=1 SV=1 – α2 chain (R) | Swissprot / [HPT_HUMAN](http://www.matrixscience.com/cgi/protein_view.pl?file=../data/20091116/FtmcobunR.dat&hit=HPT_HUMAN&db_idx=1&px=1&ave_thresh=27&_ignoreionsscorebelow=0&report=20&_sigthreshold=0.05&_msresflags=1089&_msresflags2=2&percolate=-1&percolate_rt=0) | 163 (27) | 671,736 | 21/6.0 | 45.8/6.1 | K.NYYKLR.T K.LRTEGDGVYTLNNEK.Q R.TEGDGVYTLNNEK.Q R.TEGDGVYTLNNEKQWINK.A | 6 | Haeme binding protein |
|  | **Haptoglobin OS=Homo sapiens GN=HP PE=1 SV=1 α2 chain (M)** | **Swissprot** [**HPT_HUMAN**](http://www.matrixscience.com/cgi/protein_view.pl?file=../data/20091116/FtmcobunR.dat&hit=HPT_HUMAN&px=1&ave_thresh=27&_sigthreshold=0.05&_server_mudpit_switch=0.001) | **212 (35)** | **555,724** | **20/5.9** | **45.8/6.1** | **K.NYYKLR.T K.LRTEGDGVYTLNNEK.Q R.TEGDGVYTLNNEK.Q R.TEGDGVYTLNNEKQWINK.A** | **6** | **Haeme binding protein** |
|  | Haptoglobin OS=Homo sapiens GN=HP PE=1 SV=1 **α2 chain (L)** | Swissprot / [HPT_HUMAN](http://www.matrixscience.com/cgi/protein_view.pl?file=../data/20091116/Ftmcobsee.dat&hit=HPT_HUMAN&px=1&ave_thresh=27&_sigthreshold=0.05&_server_mudpit_switch=0.001) | 168 (27) | 450,730 | 21/5.5 | 45.8/6.1 | **K.LRTEGDGVYTLNNEK.Q R.TEGDGVYTLNNEK.Q R.TEGDGVYTLNNEKQWINK.A** | 4 | Haeme binding protein |
|  | Lacrimal lipocalin precursor | MSDB/ [LCHUL](http://www.matrixscience.com/cgi/protein_view.pl?file=../data/20080514/FtgpIbeTh.dat&hit=LCHUL&px=1&ave_thresh=36&_sigthreshold=0.05&_server_mudpit_switch=0.001) | 394 (35) | 406,750 | 18/5.3 | 19.2/5.39 | **K.VTMLISGR.C K.DHYIFYCEGELHGKPVR.G K.NNLEALEDFEK.A K.NNLEALEDFEKAAGAR.G R.GLSTESILIPR.Q** | 29 | Lipid scavenging and transport to outer tear layer |
|  | Human tear lipocalin | NCBI/ [gi\|56554584](http://www.matrixscience.com/cgi/protein_view.pl?file=../data/20060606/FAGtrauO.dat&hit=1) | 72 (64) | 340,731 | 18.3/4.76 | 17.9/5.27 | **R.EFPEMNLESVTPMTLTTLEGGNLEAK.V**  **K.HVAYIIR.S K.NNLEALEDFEK.A R.GLSTESILIPR.Q** | 33 | Lipid scavenging and transport to outer tear layer |
|  | Prolactin inducible protein | NCBI/ [gi\|4505821](http://www.matrixscience.com/cgi/protein_view.pl?file=../data/20060623/FAGrIrSE.dat&hit=1) | 92 (64) | 244,727 | 20/4.24 | 16.5/8.2 | **K.SVRPNDEVTAVLAVQTELK.E**  **K.TYLISSIPLQGAFNYK.Y**  **K.YTACLCDDNPK.T R.TVQIAAVVDVIR.E R.ELGICPDDAAVIPIK.N R.FYTIEILK.V** | 55 | Suppressing T-cell apoptosis |
|  | **Lacrimal lipocalin precursor** | **MSDB/** [**LCHUL**](http://www.matrixscience.com/cgi/protein_view.pl?file=../data/20080514/FtgpIbsaT.dat&hit=LCHUL&px=1&ave_thresh=36&_sigthreshold=0.05&_server_mudpit_switch=0.001) | **112 (36)** | **174,733** | **19/4.4** | **19.2/5.39** | **K.NNLEALEDFEK.A R.GLSTESILIPR.Q** | **12** | **Lipid scavenging and transport to outer tear layer** |
|  | Full putative lipocalin 1 like protein | Swissprot/ [LC1L1_HUMAN](http://www.matrixscience.com/cgi/protein_view.pl?file=../data/20110222/FttolzaOO.dat&hit=LC1L1_HUMAN&db_idx=1&px=1&ave_thresh=30&_ignoreionsscorebelow=0&report=20&_sigthreshold=0.05&_msresflags=1089&_msresflags2=2&percolate=-1&percolate_rt=0) | 85 (28) | 121,756 | 20/4.8 | 17.9/4.93 | **R.GLSTESILIPR.Q** | 6 | Lipid scavenging and transport to outer tear layer |
|  | Prolactin-inducible protein | Swissprot/[PIP_HUMAN](http://www.matrixscience.com/cgi/protein_view.pl?file=../data/20110222/FttolzsmE.dat&hit=PIP_HUMAN&db_idx=1&px=1&ave_thresh=29&_ignoreionsscorebelow=0&report=20&_sigthreshold=0.05&_msresflags=1089&_msresflags2=2&percolate=-1&percolate_rt=0) | 546 (29) | 174,785 | 20/4.8 | 16.8/8.2 | **R.KIIIKNFDIPK.S K.IIIKNFDIPK.S K.NFDIPK.S K.SVRPNDEVTAVLAVQTELKECMVVK.T K.TYLISSIPLQGAFNYK.Y K.YTACLCDDNPK.T R.TVQIAAVVDVIR.E R.ELGICPDDAAVIPIK.N R.ELGICPDDAAVIPIKNNR.F K.NNRFYTIEILKVE R.FYTIEILK.V R.FYTIEILKVE** | 70 | Suppressing T-cell apoptosis |
|  | hCG 201503 | NCBI/[gi\|119608459](http://www.matrixscience.com/cgi/protein_view.pl?file=../data/20110826/FtoAibTaT.dat&hit=gi%7c119608459&db_idx=1&px=1&ave_thresh=38&_ignoreionsscorebelow=0&report=20&_sigthreshold=0.05&_msresflags=1025&_msresflags2=2&percolate=-1&percolate_rt=0) | 160 (26) | 195,781 | 20/4.8 | 19.3/5.2 | **K.HVAYIIR.S K.LVGRDPENNLEALEDFEK.A R.GLSTESILIPR.Q R.GLSTESILIPRQSETCSPGSD.-** | 26 | unknown |
|  | PIP precursor | NCBI/[gi\|4505821](http://www.matrixscience.com/cgi/protein_view.pl?file=../data/20110222/FttolzuOL.dat&hit=gi%7c4505821&db_idx=1&px=1&ave_thresh=34&_ignoreionsscorebelow=0&report=20&_sigthreshold=0.05&_msresflags=1089&_msresflags2=2&percolate=-1&percolate_rt=0) | 134 (32) | 221,783 | 20/5.2 | 16.5/8.2 | **K.NFDIPK.S K.YTACLCDDNPK.T R.TVQIAAVVDVIR.E R.ELGICPDDAAVIPIK.N R.FYTIEILK.V** | 35 | Suppressing T-cell apoptosis |
|  | Ig Kappa 1 light chain | NCBI/ [gi\|170684404](http://www.matrixscience.com/cgi/protein_view.pl?file=../data/20110222/FttolzuSt.dat&hit=gi%7c170684404&db_idx=1&px=1&ave_thresh=37&_ignoreionsscorebelow=0&report=20&_sigthreshold=0.05&_msresflags=1089&_msresflags2=2&percolate=-1&percolate_rt=0) | 163 (37) | 240,788 | 20/5.2 | 23.5/6.08 | **R.TVAAPSVFIFPPSDEQLK.S K.DSTYSLSSTLTLSK.A K.VYACEVTHQGLSSPVTK.S** | 22 | Antigen Binding |
|  | **Lacritin Precursor** | **NCBI/** [**gi\|15187164**](http://www.matrixscience.com/cgi/protein_view.pl?file=../data/20080514/FtgpIeeaT.dat&hit=gi%7c15187164&px=1&ave_thresh=38&_sigthreshold=0.05&_server_mudpit_switch=0.001) | **62 (38)** | **244,622** | **26/4.8** | **14.2/5.43** | **K.SILLTEQALAK.A K.KFSLLKPWA** | **14** | **Secretion, renewal of lacrimal & ocular surface epithelia** |
|  | Lacritin precursor | NCBI/ [gi\|15187164](http://www.matrixscience.com/cgi/protein_view.pl?file=../data/20080514/FtgpIeeaT.dat&hit=gi%7c15187164&px=1&ave_thresh=38&_sigthreshold=0.05&_server_mudpit_switch=0.001) | 62 (38) | 373,627 | 26/5.3 | 14.2/5.4 | **K.SILLTEQALAK.A K.KFSLLKPWA.-** | 14 | Secretion, renewal of lacrimal & ocular surface epithelia |
|  | **Pro Apolipoprotein** | **NCBI/**[**gi\|178775**](http://www.matrixscience.com/cgi/protein_view.pl?file=../data/20110222/FttolzamO.dat&hit=gi%7c178775&db_idx=1&px=1&ave_thresh=38&_ignoreionsscorebelow=0&report=20&_sigthreshold=0.05&_msresflags=1025&_msresflags2=2&percolate=-1&percolate_rt=0) | **204 (27)** | **367,575** | **30/5.2** | **28.9/5.45** | **K.LLDNWDSVTSTFSK.L R.THLAPYSDELRQR.L K.ATEHLSTLSEK.A K.AKPALEDLR.Q** | **18** | **Lipid profile regulator** |
|  | Chain A, Crystal Structure Of Lipid-Free Human ApolipoproteinA-I | NCBI / [gi\|90108664](http://www.matrixscience.com/cgi/protein_view.pl?file=../data/20091117/FtmcmieaE.dat&hit=gi%7c90108664&px=1&ave_thresh=36&_sigthreshold=0.05&_server_mudpit_switch=0.001) | 507 (36) | 399,574 | 29/5.1 | 28/5.2 | **-.DEPPQSPWDRVK.D R.DYVSQFEGSALGK.Q K.LLDNWDSVTSTFSK.L R.QEMSKDLEEVK.A K.VEPLRAELQEGAR.Q R.QKLHELQEK.L K.LSPLGEEMRDR.A R.ARAHVDALR.T R.THLAPYSDELRQR.L K.ATEHLSTLSEK.A K.AKPALEDLR.Q** | 51 | Lipid profile regulator |
|  | Ig alpha | Swissprot/ [IGHA1_HUMAN](http://www.matrixscience.com/cgi/protein_view.pl?file=../data/20091116/FtmcobeTE.dat&hit=IGHA1_HUMAN&px=1&ave_thresh=28&_sigthreshold=0.05&_server_mudpit_switch=0.001) | 86 (28) | 749,241 | 38.4/6.08 | 45/6.2 | **K.TPLTATLSK.S R.EKYLTWASR.Q K.YLTWASR.Q** | 5 | Major Immunoglobulin Class In Body Secretions |
|  | apolipoprotein E [Homo sapiens] | NCBI/[gi\|178849](http://www.matrixscience.com/cgi/protein_view.pl?file=../data/20110826/FtoAibTTE.dat&hit=gi%7c178849&db_idx=1&px=1&ave_thresh=38&_ignoreionsscorebelow=0&report=20&_sigthreshold=0.05&_msresflags=1025&_msresflags2=2&percolate=-1&percolate_rt=0) | 124(37) | 509,462 | 32/5.5 | 36.1/5.65 | **R.LGPLVEQGR.V R.AKLEEQAQQIR.L R.LQAEAFQAR.L** | 9 | Lipid profile regulator |
|  | Lacrimal lipocalin precursor | MSDB/ [LCHUL](http://www.matrixscience.com/cgi/protein_view.pl?file=../data/20080514/FtgpIbSmm.dat&hit=LCHUL&px=1&ave_thresh=36&_sigthreshold=0.05&_server_mudpit_switch=0.001) | 142 (36) | 431,394 | 40/5.1 | 19.2/5.39 | **K.NNLEALEDFEK.A R.GLSTESILIPR.Q** | 12 | Lipid scavenging and transport to outer tear layer |
|  | \|  \|  \| \| --- \| --- \|   Zn-alpha2-glycoprotein [Homo sapiens] | NCBI/[gi\|38026](http://www.matrixscience.com/cgi/protein_view.pl?file=../data/20100703/FteoSzEOO.dat&hit=gi%7c38026&db_idx=1&px=1&ave_thresh=37&_ignoreionsscorebelow=0&report=20&_sigthreshold=0.05&_msresflags=1025&_msresflags2=2&percolate=-1&percolate_rt=0) | 86 (37) | 249,307 | 45/4.6 | 34.7/5.7 | K.EIPAWVPFDPAAQITK.Q K.AYLEEECPATLR.K | 9 | Stimulates lipid degradation |
|  | **Zn-alpha2-glycoprotein [Homo sapiens]** | **NCBI/**[**gi\|38026**](http://www.matrixscience.com/cgi/protein_view.pl?file=../data/20080514/FtgpIesEe.dat&hit=gi%7c38026&px=1&ave_thresh=38&_sigthreshold=0.05&_server_mudpit_switch=0.001) | **607 (36)** | **293,317** | **51/4.8** | **34.7/5.71** | **R.YSLTYIYTGLSK.H K.SQPMGLWR.Q R.QVEGMEDWK.Q R.QVEGMEDWKQDSQLQK.A K.AREDIFMETLK.D R.EDIFMETLK.D K.YYYDGKDYIEFNK.E K.QKWEAEPVYVQR.A K.WEAEPVYVQR.A K.AYLEEECPATLR.K K.AYLEEECPATLRK.Y R.QDPPSVVVTSHQAPGEK.K K.CLAYDFYPGK.I** | **37** | **Stimulates lipid degradation** |
|  | Zinc-alpha-2-glycoprotein OS=Homo sapiens | NCBI/[gi\|38026](http://www.matrixscience.com/cgi/protein_view.pl?file=../data/20110826/FtoAibTet.dat&hit=gi%7c38026&db_idx=1&px=1&ave_thresh=35&_ignoreionsscorebelow=0&report=20&_sigthreshold=0.05&_msresflags=1025&_msresflags2=2&percolate=-1&percolate_rt=0) | 134(35) | 332,317 | 39/5.3 | 34.7/5.7 | **R.YSLTYIYTGLSK.H R.SSGAFWK.Y R.AGEVQEPELR.G** | 9 | Stimulates lipid degradation |
|  | **Haptoglobin precursor – β chain** | **NCBI/**[**gi\|306882**](http://www.matrixscience.com/cgi/protein_view.pl?file=../data/20110826/FtoAibTeR.dat&hit=gi%7c306882&db_idx=1&px=1&ave_thresh=35&_ignoreionsscorebelow=0&report=20&_sigthreshold=0.05&_msresflags=1025&_msresflags2=2&percolate=-1&percolate_rt=0) | **182(35)** | **379,336** | **39/5.6** | **45.1/6.24** | **R.ILGGHLDAK.G K.GSFPWQAK.M K.DIAPTLTLYVGK.KK.QLVEIEK.V R.VGYVSGWGR.N K.FTDHLK.Y K.VTSIQDWVQK.T** | **15** | **Haeme binding protein** |
|  | Zinc-alpha-2-glycoprotein, chain A - human | MSDB/[1ZAGA](http://www.matrixscience.com/cgi/protein_view.pl?file=../data/20080514/FtgpIzEaL.dat&hit=1ZAGA&px=1&ave_thresh=36&_sigthreshold=0.05&_server_mudpit_switch=0.001) | 213 (36) | 407,353 | 45/5.4 | 31.5/5.70 | **R.YSLTYIYTGLSK.H K.YYYDGKDYIEFNK.E K.AYLEEECPATLR.K K.CLAYDFYPGK.I** | 17 | Stimulates lipid degradation |
|  | Actin, cytoplasmic 1 | Swissprot/ [ACTB_HUMAN](http://www.matrixscience.com/cgi/protein_view.pl?file=../data/20091117/FtmcmiaSS.dat&hit=ACTB_HUMAN&px=1&ave_thresh=28&_sigthreshold=0.05&_server_mudpit_switch=0.001) | 94 (28) | 397,291 | 39/5.5 | 42/5.29 | **K.AGFAGDDAPR.A R.GYSFTTTAER.E K.IIAPPER.K K.IIAPPERK.Y** | 7 | Structural protein |
|  | Haptoglobin isoform 2 pre protein -  **β chain** | NCBI/[gi\|186910296](http://www.matrixscience.com/cgi/protein_view.pl?file=../data/20110826/FtoAibTmR.dat&hit=gi%7c186910296&db_idx=1&px=1&ave_thresh=38&_ignoreionsscorebelow=0&report=20&_sigthreshold=0.05&_msresflags=1025&_msresflags2=2&percolate=-1&percolate_rt=0) | 234(38) | 430,342 | 40/5.7 | 38.4/6.1 | **K.GSFPWQAK.M K.DIAPTLTLYVGKK.Q K.QLVEIEK.V K.YVMLPVADQDQCIR.H K.SCAVAEYGVYVK.V K.VTSIQDWVQK.T** | 18 | Haeme binding protein |
|  | **lactoferrin [Homo sapiens]** | NCBI/[gi\|2104522](http://www.matrixscience.com/cgi/protein_view.pl?file=../data/20100626/FteofxYEh.dat&hit=gi%7c2104522&db_idx=1&px=1&ave_thresh=38&_ignoreionsscorebelow=0&report=20&_sigthreshold=0.05&_msresflags=1025&_msresflags2=2&percolate=-1&percolate_rt=0) | **79 (38)** | **485,337** | 42/5.6 | **52.3/7** | **R.KSEEEVAAR.R**   **K.CGLVPVLAENYK.S**  **R.CLAENAGDVAFVK.D**  **R.KPVTEAR.S** | **8** | Antimicrobial activity |
|  | **ALB protein (Growth-inhibiting protein 20).- Homo sapiens (Human).** | MSDB/[Q86YG0_HUMAN](http://www.matrixscience.com/cgi/protein_view.pl?file=../data/20100628/Fteoractm.dat&hit=Q86YG0_HUMAN&db_idx=1&px=1&ave_thresh=36&_ignoreionsscorebelow=0&report=20&_sigthreshold=0.05&_msresflags=1089&_msresflags2=2&percolate=-1&percolate_rt=0) | **532(36)** | **619,357** | 45/6.1 | **47.3/5.9** | **K.AWAVAR.L**  **R.LSQRFPK.A R.LSQRFPK.A K.AEFAEVSK.L**  **K.LVTDLTK.V**  **K.VHTECCHGDLLECADDRADLAK.Y**  **K.LKECCEKPLLEK.S**  **K.LKECCEKPLLEK.S**  **K.TYETTLEK.C**  **K.CCAAADPHECYAK.V** **K.VFDEFKPLVEEPQNLIK.Q**  **K.QNCELFEQLGEYK.F**  **K.FQNALLVR.Y**  **K.FQNALLVR.Y**  **K.KVPQVSTPTLVEVSR.N**  **K.CCKHPEAK.R**  **K.CCKHPEAK.R**  **K.HPEAKR.M**  **K.TPVSDRVTK.C**  **K.CCTESLVNR.R**  **R.RPCFSALEVDETYVPK.E**  **K.KQTALVELVK.H**  **K.KQTALVELVK.H**  **K.QTALVELVK.H**  **K.QTALVELVK.H**  **K.AVMDDFAAFVEK.C**  **K.AVMDDFAAFVEK.C**  **K.LVAASQAALGL.-** | **50** | Regulation of the colloidal osmotic pressure of blood |
|  | \|  \| **albumin-like [Homo sapiens** \| \| --- \| --- \| | NCBI/[gi\|763431](http://www.matrixscience.com/cgi/protein_view.pl?file=../data/20100628/FteoraHSR.dat&hit=gi%7c763431&db_idx=1&px=1&ave_thresh=37&_ignoreionsscorebelow=0&report=20&_sigthreshold=0.05&_msresflags=1025&_msresflags2=2&percolate=-1&percolate_rt=0)**]** | **206(37)** | **730,293** | 50/6.8 | **52/5.6** | **K.LVNEVTEFAK.T K.LVNEVTEFAK.T K.YLYEIAR.R K.LVTDLTK.V K.LVTDLTK.V K.VHTECCHGDLLECADDRADLAK.YK.VHTECCHGDLLECADDRADLAK.Y**  **R.RHPDYSVVLLLR.L K.QNCELFEQLGEYK.F** | **15** | Regulation of the colloidal osmotic pressure of blood |
|  | **ALB protein [Homo sapiens]** | \|  \| NCBI/[gi\|27692693](http://www.matrixscience.com/cgi/protein_view.pl?file=../data/20100626/FteofxYwT.dat&hit=gi%7c27692693&db_idx=1&px=1&ave_thresh=39&_ignoreionsscorebelow=0&report=20&_sigthreshold=0.05&_msresflags=1025&_msresflags2=2&percolate=-1&percolate_rt=0) \| \| --- \| --- \| | **162(39)** | **545,273** | 49/5.8 | **47.3/5.9** | **R.LSQRFPK.A K.LVTDLTK.V K.LVTDLTK.V K.KVPQVSTPTLVEVSR.N K.KVPQVSTPTLVEVSR.N K.KQTALVELVK.H** | **9** | Regulation of the colloidal osmotic pressure of blood |
|  | **ALB protein [Homo sapiens]** | NCBI/[gi\|27692693](http://www.matrixscience.com/cgi/protein_view.pl?file=../data/20100626/FteofzYES.dat&hit=gi%7c27692693&db_idx=1&px=1&ave_thresh=39&_ignoreionsscorebelow=0&report=20&_sigthreshold=0.05&_msresflags=1089&_msresflags2=2&percolate=-1&percolate_rt=0) | **513(39)** | **569,318** | 44/5.9 | **47.3/5.9** | **K.AWAVAR.L R.LSQRFPK.A**  **K.AEFAEVSK.L**  **K.LVTDLTK.V K.VHTECCHGDLLECADDRADLAK.Y K.LKECCEKPLLEK.S**  **K.TYETTLEK.C**  **K.CCAAADPHECYAK.V**  **K.VFDEFKPLVEEPQNLIK.Q**  **K.QNCELFEQLGEYK.F**  **K.FQNALLVR.Y**  **K.KVPQVSTPTLVEVSR.N**  **K.CCKHPEAK.R K.HPEAKR.M**  **K.TPVSDRVTK.C**  **K.CCTESLVNR.R**  **R.RPCFSALEVDETYVPK.E**  **K.KQTALVELVK.H**  **K.QTALVELVK.H**  **K.AVMDDFAAFVEK.C**  **K.LVAASQAALGL.-** | 50 | Regulation of the colloidal osmotic pressure of blood |
|  | Serum albumin OS=Homo sapiens GN=ALB PE=1 SV=2 | Swissprot/[ALBU_HUMAN](http://www.matrixscience.com/cgi/protein_view.pl?file=../data/20110826/FtoAibTSR.dat&hit=ALBU_HUMAN&db_idx=1&px=1&ave_thresh=29&_ignoreionsscorebelow=0&report=20&_sigthreshold=0.05&_msresflags=1089&_msresflags2=2&percolate=-1&percolate_rt=0) | 519(29) | 644,228 | 66/6 | 69.3/5.92 | **K.LVNEVTEFAK.T K.SLHTLFGDK.L K.LCTVATLR.E R.NECFLQHK.D K.DDNPNLPR.L K.KYLYEIAR.R K.YLYEIAR.R K.AACLLPK.L K.LDELRDEGK.A K.AWAVAR.L**  **R.LSQRFPK.A K.AEFAEVSK.L K.LVTDLTK.V R.HPDYSVVLLLR.LK.TYETTLEK.C K.FQNALLVR.Y K.CCTESLVNR.R K.KQTALVELVK.H K.QTALVELVK.H K.AVMDDFAAFVEK.C K.KLVAASQAALGL.-K.LVAASQAALGL.-** | 27 | Regulation of the colloidal osmotic pressure of blood |
|  | Ig alpha-1 chain C region | Swissprot/ [IGHA1_HUMAN](http://www.matrixscience.com/cgi/protein_view.pl?file=../data/20091116/FtmcobeTE.dat&hit=IGHA1_HUMAN&px=1&ave_thresh=28&_sigthreshold=0.05&_server_mudpit_switch=0.001) | 86 (28) | 741,237 | 38.4/6.08 | 45/6.2 | **K.TPLTATLSK.S R.EKYLTWASR.Q K.YLTWASR.Q** | 5 | Major Immunoglobulin Class In Body Secretions |
|  | Ig gamma-1 chain C region OS=Homo sapiens GN=IGHG1 PE=1 SV=1 | Swissprot/[IGHG1_HUMAN](http://www.matrixscience.com/cgi/protein_view.pl?file=../data/20110826/FtoAibTEm.dat&hit=IGHG1_HUMAN&db_idx=1&px=1&ave_thresh=30&_ignoreionsscorebelow=0&report=20&_sigthreshold=0.05&_msresflags=1089&_msresflags2=2&percolate=-1&percolate_rt=0) | 89(28) | 756,235 | 43/6.2 | 36/8.46 | **K.GPSVFPLAPSSK.S K.DTLMISR.T K.ALPAPIEK.T K.NQVSLTCLVK.G** | 11 | Major Immunoglobulin Class In Body Secretions |
|  | **Lactoferrin** | **NCBI/**[**gi\|2104522**](http://www.matrixscience.com/cgi/protein_view.pl?file=../data/20100311/FtmTlfSnS.dat&hit=gi%7c2104522&px=1&ave_thresh=38&_sigthreshold=0.05&_server_mudpit_switch=0.001) | **222 (38)** | **800,269** | **50/7** | **53.6/7.09** | **K.CGLVPVLAENYK.S R.SDTSLTWNSVK.G R.CLAENAGDVAFVK.D** | **9** | **Antimicrobial activity** |
|  | Lactoferrin | NCBI/[gi\|2104522](http://www.matrixscience.com/cgi/protein_view.pl?file=../data/20100311/FtmTlfHTS.dat&hit=gi%7c2104522&px=1&ave_thresh=39&_sigthreshold=0.05&_server_mudpit_switch=0.001) | 155 (28) | 834,218 | 50/7 | 53.6/7 | **K.CGLVPVLAENYK.S R.SDTSLTWNSVK.G R.CLAENAGDVAFVK.D** | 7 | Antimicrobial activity |
|  | \|  \| RecName: Full=Ig gamma-4 chain C region \| \| --- \| --- \| | NCBI/[gi\|121047](http://www.matrixscience.com/cgi/protein_view.pl?file=../data/20100702/FteoSrEwE.dat&hit=gi%7c121047&db_idx=1&px=1&ave_thresh=36&_ignoreionsscorebelow=0&report=20&_sigthreshold=0.05&_msresflags=1025&_msresflags2=2&percolate=-1&percolate_rt=0) | 111(36) | 793,177 | 70/7 | 35.9/7.1 | **K.GPSVFPLAPCSR.S R.STSESTAALGCLVK.D K.NQVSLTCLVK.G K.NQVSLTCLVK.G** | **11** | Major Immunoglobulin Class In Body Secretions |
|  | Chain A, Structure Of Human Apolactoferrin At 2.0 A Resolution | NCBI/[gi\|4699853](http://www.matrixscience.com/cgi/protein_view.pl?file=../data/20100702/FteoSfcTt.dat&hit=gi%7c4699853&db_idx=1&px=1&ave_thresh=38&_ignoreionsscorebelow=0&report=20&_sigthreshold=0.05&_msresflags=1025&_msresflags2=2&percolate=-1&percolate_rt=0) | **105(38)** | **712,219** | 67/6.8 | **76.1/8.4** | **R.DGAGDVAFIR.E K.DLLFK.D R.CLAENAGDVAFVK.D** | **4** | Antimicrobial activity |
|  | Serotransferrin precursor [Homo sapiens] | NCBI/[gi\|4557871](http://www.matrixscience.com/cgi/protein_view.pl?file=../data/20100702/FteoSfuTR.dat&hit=gi%7c4557871&db_idx=1&px=1&ave_thresh=38&_ignoreionsscorebelow=0&report=20&_sigthreshold=0.05&_msresflags=1025&_msresflags2=2&percolate=-1&percolate_rt=0) | **196(38)** | **668,181** | 68/6.6 | **77/6.8** | **K.SVIPSDGPSVACVK.K K.SASDLTWDNLK.G R.FDEFFSEGCAPGSK.K K.EGYYGYTGAFR.C** | **7** | iron-binding blood plasma glycoproteins |
|  | transferrin precursor [validated] - human | MSDB/[TFHUP](http://www.matrixscience.com/cgi/protein_view.pl?file=../data/20100702/FteoSfYeR.dat&hit=TFHUP&db_idx=1&px=1&ave_thresh=35&_ignoreionsscorebelow=0&report=20&_sigthreshold=0.05&_msresflags=1025&_msresflags2=2&percolate=-1&percolate_rt=0) | **1568(35)** | **573,82** | 66/6.3 | **77/6.8** | **K.SVIPSDGPSVACVKK.A K.ASYLDCIR.A K.DSGFQMNQLR.G K.CLKDGAGDVAFVK.H K.DGAGDVAFVK.H K.HSTIFENLANK.A K.DCHLAQVPSHTVVAR.S K.EFQLFSSPHGK.D K.MYLGYEYVTAIR.N K.CDEWSVNSVGKIECVSAETTEDCIAK.I K.IECVSAETTEDCIAK.I K.KSASDLTWDNLK.G K.SASDLTWDNLK.G R.TAGWNIPMGLLYNK.I R.FDEFFSEGCAPGSK.K K.LCMGSGLNLCEPNNK.E K.EGYYGYTGAFR.C R.CLVEKGDVAFVK.H K.NLNEKDYELLCLDGTR.K K.DLLFR.D K.DLLFRDDTVCLAK.L K.YLGEEYVK.A R.KCSTSSLLEACTFR.R K.CSTSSLLEACTFR.R** | **35** | iron-binding blood plasma glycoproteins |
|  | **Serum albumin, chain A** | **MSDB /** [**1AO6A**](http://www.matrixscience.com/cgi/protein_view.pl?file=../data/20080514/FtgpIesnO.dat&hit=1AO6A&px=1&ave_thresh=36&_sigthreshold=0.05&_server_mudpit_switch=0.001) | **639 (36)** | **550,131** | **66/5.1** | **65.6/5.63** | **R.FKDLGEENFK.A K.LVNEVTEFAK.T K.KYLYEIAR.R K.YLYEIAR.R K.VHTECCHGDLLECADDR.A K.VHTECCHGDLLECADDRADLAK.Y K.QNCELFEQLGEYK.F K.FQNALLVR.Y K.KVPQVSTPTLVEVSR.N K.VPQVSTPTLVEVSR.N K.QTALVELVK.H K.AVMDDFAAFVEK.C** | **18** | **Regulation of the colloidal osmotic pressure of blood** |
|  | Chain A, Human Serum Albumin In A Complex With Myristic Acid And Tri- Iodobenzoic Acid | NCBI/[gi\|157830361](http://www.matrixscience.com/cgi/protein_view.pl?file=../data/20110826/FtoAibTSO.dat&hit=gi%7c157830361&db_idx=1&px=1&ave_thresh=38&_ignoreionsscorebelow=0&report=20&_sigthreshold=0.05&_msresflags=1089&_msresflags2=2&percolate=-1&percolate_rt=0) | 917(27) | 467,135 | 66/5.6 | 65.9/5.69 | **R.FKDLGEENFK.A K.LVNEVTEFAK.T K.SLHTLFGDK.L K.LCTVATLR.E R.ETYGEMADCCAK.Q K.KYLYEIAR.R K.YLYEIAR.R K.YLYEIARR.H K.AAFTECCQAADK.A K.AACLLPK.L K.YICENQDSISSK.L K.LKECCEKPLLEK.S K.ECCEKPLLEK.S K.FQNALLVR.Y K.KVPQVSTPTLVEVSR.N K.VPQVSTPTLVEVSR.N R.RPCFSALEVDETYVPK.E K.KQTALVELVK.H K.QTALVELVK.H K.LVAASQAALG.-** | 27 | Regulation of the colloidal osmotic pressure of blood |
|  | Unnamed protein | NCBI / [gi\|34412](http://www.matrixscience.com/cgi/protein_view.pl?file=../data/20100310/FtmTieYmL.dat&hit=gi%7c34412&px=1&ave_thresh=38&_sigthreshold=0.05&_server_mudpit_switch=0.001) | 162 (37) | 432,192 | 66/ 6.1 | 79.9/ 8.5 | **R.VVWCAVGEQELRK.C K.CGLVPVLAENYK.S R.CLAENAGDVAFVK.D** | 5 | Unknown |
|  | Albumin, isoform CRA H | NCBI/[gi\|119626071](http://www.matrixscience.com/cgi/protein_view.pl?file=../data/20110826/FtoAibetL.dat&hit=gi%7c119626071&db_idx=1&px=1&ave_thresh=38&_ignoreionsscorebelow=0&report=20&_sigthreshold=0.05&_msresflags=1089&_msresflags2=2&percolate=-1&percolate_rt=0) | 569(28) | 397,195 | 66/5.5 | 68.5/5.92 | **R.FKDLGEENFK.A K.LVNEVTEFAK.T K.SLHTLFGDK.L K.LCTVATLR.E R.ETYGEMADCCAK.Q K.KYLYEIAR.R K.YLYEIAR.R K.YLYEIARR.H K.AAFTECCQAADK.A K.AACLLPK.L K.YICENQDSISSK.L K.LKECCEKPLLEK.S K.ECCEKPLLEK.S**  **K.FQNALLVR.Y K.VPQVSTPTLVEVSR.N K.KQTALVELVK.H K.QTALVELVK.H K.KLVAASQAALGL.-K.LVAASQAALGL.-** | 24 | Regulation of the colloidal osmotic pressure of blood |
|  | Chain A, Human serum albumin complex with myristic acid and tri iodo benzene acid | Swissprot/[gi\|157830361](http://www.matrixscience.com/cgi/protein_view.pl?file=../data/20110826/FtoAibeaR.dat&hit=gi%7c157830361&db_idx=1&px=1&ave_thresh=35&_ignoreionsscorebelow=0&report=20&_sigthreshold=0.05&_msresflags=1089&_msresflags2=2&percolate=-1&percolate_rt=0) | 371(27) | 366,199 | 66/5.4 | 65.9/5.69 | **K.DLGEENFK.A K.LVNEVTEFAK.T K.SLHTLFGDK.L K.LCTVATLR.E**  **K.YLYEIAR.R K.AACLLPK.L K.ECCEKPLLEK.S R.HPDYSVVLLLR.L K.FQNALLVR.Y K.QTALVELVK.H K.AVMDDFAAFVEK.C K.LVAASQAALG.-** | 18 | Regulation of the colloidal osmotic pressure of blood |
|  | **Alpha-1-antitrypsin** | **Swissprot /**[**A1AT_HUMAN**](http://www.matrixscience.com/cgi/protein_view.pl?file=../data/20091117/FtmcmfYTm.dat&hit=A1AT_HUMAN&db_idx=1&px=1&ave_thresh=28&_ignoreionsscorebelow=0&report=20&_sigthreshold=0.05&_msresflags=1089&_msresflags2=2&percolate=-1&percolate_rt=0) | **76 (28)** | **269,210** | **45/5.2** | **46.8/5.3** | **K.TDTSHHDQDHPTFNK.I K.IVDLVK.E K.FLENEDRR.S K.LSITGTYDLK.S** | **9** | **Inhibitor of serine proteases** |
|  | Alpha-1-antitrypsin | Swissprot / [A1AT_HUMAN](http://www.matrixscience.com/cgi/protein_view.pl?file=../data/20091117/FtmcmfYTm.dat&hit=A1AT_HUMAN&db_idx=1&px=1&ave_thresh=28&_ignoreionsscorebelow=0&report=20&_sigthreshold=0.05&_msresflags=1089&_msresflags2=2&percolate=-1&percolate_rt=0) | 76 (28) | 244,206 | 45/5.1 | 46.8/5.3 | **K.TDTSHHDQDHPTFNK.I K.IVDLVK.E K.FLENEDRR.S K.LSITGTYDLK.S** | 9 | Inhibitor of serine proteases |

*^a^*MOWSE scores greater than the values given in the parenthesis are considered to be significant (*p* < 0.05). All proteins were also searched across multiple databases to confirm their identity. *^b^*Apparent/ experimental molecular weight and pI of protein spot on 2-DE gels. *^c^*Theoretical molecular weight and p*I* of the identified protein in database. *^d^*Represents the peptides matched. ^e^Sequence coverage (SC) represents the % aminoacid sequence covered in the protein by the matched peptides. 18R, 19M and 20L of α2 isoforms are labeled according to Gupta [22] *et al.,*2007. Spot 5 is of very low intensity and spot 33 is a highly abundant protein. Under our conditions both give vey high MOWSE scores.
