# Supplementary material for: Pathogen Induced Changes in the Protein Profile of Human Tears from Fusarium Keratitis Patients
Source: PLoS One. 2013 Jan 8;8(1):e53018. doi: 10.1371/journal.pone.0053018 (PMC3540078; doi:10.1371/journal.pone.0053018)
Supplement: Table S3 — Classification of differentially regulated proteins according to biological process, cellular & molecular. (DOCX) [file pone.0053018.s003.docx]

| **Cellular Component** | |
| --- | --- |
| **Category** | **Gene ID** |
| Extracellular region | **↓**LCN1, **↑**AZGP1, **↑**APOA2, **↑**ALB, **↓**CST4,**↓** LACRT, **↑**LTF, **↑**HP, **↑**SERPINA1 |
| Spherical high-density lipoprotein particle | **↑**APOA2,**↑** HP |
| Secretory granule | **↑**ALB, **↓**LACRT, **↑**SERPINA1 |
| Extracellular space | **↑**APOA2,**↑** ALB, **↑**HP, **↑**SERPINA1 |
| High-density lipoprotein particle | **↑**APOA2,**↑** HP |
| Extracellular region part | **↑**APOA2, **↑**ALB, **↑**HP, **↑**SERPINA1 |
| Plasma lipoprotein particle | **↑**APOA2,**↑** HP |
| Protein-lipid complex | **↑**APOA2,**↑** HP |
| Platelet alpha granule lumen | **↑**ALB, **↑**SERPINA1 |
| Cytoplasmic membrane-bounded vesicle lumen | **↑**ALB,**↑** SERPINA1 |
| Vesicle lumen | **↑**ALB, **↑**SERPINA1 |
| Platelet alpha granule | **↑**ALB, **↑**SERPINA1 |
| Cytoplasmic membrane-bounded vesicle | **↑**ALB, **↓**LACRT,**↑** SERPINA1 |
| Membrane-bounded vesicle | **↑**ALB, **↓**LACRT, **↑**SERPINA1 |
| Cytoplasmic vesicle | **↑**ALB,**↓** LACRT, **↑**SERPINA1 |
| Vesicle | **↑**ALB,**↓** LACRT,**↑** SERPINA1 |

**Supplementary Table S3: Classification of differentially regulated proteins according to biological process, cellular & molecular**

**function**

| **Molecular Function** | |
| --- | --- |
| **Category** | **Gene ID** |
| Enzyme inhibitor activity | **↓**LCN1, **↑**APOA2, **↓**CST4, **↑**SERPINA1 |
| Endopeptidase inhibitor activity | **↓**LCN1, **↓**CST4, **↑**SERPINA1 |
| Peptidase inhibitor activity | **↓**LCN1, **↓** CST4, **↑**SERPINA1 |
| Cysteine-type endopeptidase inhibitor activity | **↓**LCN1, **↓**CST4 |
| Fatty acid binding | **↑**AZGP1, **↑**ALB |
| Lipid binding | **↑**AZGP1, **↑** APOA2, **↑** ALB |
| Monocarboxylic acid binding | **↑**AZGP1, **↑**ALB |
| Peptidase activity | **↑**LTF, **↑**HP, **↑**SERPINA1 |
| Carboxylic acid binding | **↑**AZGP1, **↑**ALB |
| Serine-type endopeptidase activity | **↑**LTF, **↑**HP |

| **Biological Process** | |
| --- | --- |
| **Category** | **Gene ID** |
| Defense response | **↑**APOA2, **↑**LTF, **↑**HP, **↑**SERPINA1 |
| Cellular iron ion homeostasis | **↑**LTF, **↑**HP |
| Iron ion homeostasis | **↑**LTF, **↑** HP |
| Chemical homeostasis | **↑**APOA2, **↑**LTF, **↑**HP |
| Acute inflammatory response | **↑**APOA2, **↑**SERPINA1 |
| Response to estrogen stimulus | **↑**APOA2, **↑**SERPINA1 |
| Homeostatic process | **↑**APOA2, **↑**LTF, **↑**HP |
| Regulation of protein transport | **↑**APOA2, **↓**LACRT |
| Regulation of establishment of protein localization | **↑**APOA2, **↓** LACRT |
| Regulation of protein localization | **↑**APOA2, **↓**LACRT |
| Regulation of body fluid levels | **↓**LACRT, **↑** SERPINA1 |
| Lipid catabolic process | **↑**AZGP1, **↑**APOA2 |
| Response to steroid hormone stimulus | **↑**APOA2, **↑** SERPINA1 |
| Response to bacterium | **↑**LTF, **↑**SERPINA1 |
| Regulation of secretion | **↑**APOA2, **↓**LACRT |

The pattern of regulation in tears obtained from keratitis patients were represented as ‘**↑’** for upregulation and ‘**↓’** for downregulation
