# Supplementary material for: Pathogen Induced Changes in the Protein Profile of Human Tears from Fusarium Keratitis Patients
Source: PLoS One. 2013 Jan 8;8(1):e53018. doi: 10.1371/journal.pone.0053018 (PMC3540078; doi:10.1371/journal.pone.0053018)
Supplement: Table S4 — Tear sample details used in this study. (DOCX) [file pone.0053018.s004.docx]

Supplementary Table S4: Tear sample details used in this study

| **Sample number** | **Age/Sex** | **Duration of symptom** | **Status** | **Characteristics of ulcer** | | **Sample pooling** | **Previous Treatment Details** |
| --- | --- | --- | --- | --- | --- | --- | --- |
|  |  |  |  | **Size** | **Depth** |  |  |
|  | 40/M | < 7 days | Early | 5-10 mm^2^ | Mild | Pool 1 | Antifungal & Antibacterial |
|  | 39/M | < 7 days | Early | 5-10 mm^2^ | Mild | Pool 1 | Antifungal & Antibacterial |
|  | 37/M | < 7 days | Early | < 5 mm^2^ | Superficial | Pool 1 | Antifungal & Antibacterial |
|  | 40/M | < 7 days | Early | < 5 mm^2^ | Superficial | Pool 1 | Antibacterial |
|  | 40/M | < 7 days | Early | 5-10 mm^2^ | Mild | Pool 1 | Antifungal & Antibacterial |
|  | 37/M | 7-14 days | Intermediate | < 5 mm^2^ | Superficial | Pool 2 | Antifungal & Antibacterial |
|  | 30/M | 7-14 days | Intermediate | 10-14 mm^2^ | Mild | Pool 2 | Antifungal & Antibacterial |
|  | 32/M | 7-14 days | Intermediate | 5-10 mm^2^ | Deep | Pool 2 | Antifungal & Antibacterial |
|  | 40/M | 7-14 days | Intermediate | 5-10 mm^2^ | Mild | Pool 2 | Antifungal & Antibacterial |
|  | 40/M | 7-14 days | Intermediate | 5-10 mm^2^ | Superficial | Pool 2 | Antibacterial |
|  | 47/M | >21 days | Late | < 5 mm^2^ | Deep | Pool 3 | Antifungal & Antibacterial |
|  | 42/M | 14-21 days | Late | 5-10 mm^2^ | Mild | Pool 3 | Antifungal & Antibacterial |
|  | 45/M | 14-21 days | Late | 5-10 mm^2^ | Superficial | Pool 3 | Antifungal & Antibacterial |
|  | 35/M | 14-21 days | Late | 10-14 mm^2^ | Deep | Pool 3 | Antifungal & Antibacterial |
|  | 40/M | >21 days | Late | 5-10 mm^2^ | mild | Pool 3 | Antifungal & Antibacterial |
|  | 20/M | - | Control | - | - | Pool 4 | - |
|  | 27/M | - | Control | - | - | Pool 4 | - |
|  | 21/M | - | Control | - | - | Pool 4 | - |
|  | 24/M | - | Control | - | - | Pool 4 | - |
|  | 38/M | - | Control | - | - | Pool 4 | - |
|  | 14/M | - | Control | - | - | Pool 4 | - |
|  | 18/M | - | Control | - | - | Pool 4 | - |
|  | 35/M | - | Control | - | - | Pool 4 | - |
|  | 28/M | - | Control | - | - | Pool 4 | - |
|  | 27/M | - | Control | - | - | Pool 4 | - |
|  | 24/M | - | Control | - | - | Pool 4 | - |
|  | 40/M | - | Control | - | - | Pool 4 | - |
|  | 19/M | - | Control | - | - | Pool 4 | - |
|  | 32/M | - | Control | - | - | Pool 4 | - |
|  | 38/M | - | Control | - | - | Pool 4 | - |
|  | 18/M | - | Control | - | - | Pool 4 | - |
|  | 20/M | - | Control | - | - | Pool 4 | - |
|  | 30/M | - | Control | - | - | Pool 4 | - |
|  | 16/M | - | Control | - | - | Pool 4 | - |
|  | 40/M | - | Control | - | - | Pool 4 | - |

‡ In all the cases associated other infectious ocular conditions were nil.

‡ All infected cases (1-15) were culture proven to Fusarium
